# A genome-resolved cyanobacterial consortium from the Atacama Desert reveals novel bacterial diversity, stress-resilience potential, chromatic flexibility, and photoprotection

**DOI:** 10.3389/fbioe.2026.1939861

**Published:** 2026-09-25

**Authors:** Alexandra Galetović, Jan Ortiz, Gabriel Peña, Patricio Arros, Nicolás Guerra-Leal, Camilo Berríos-Pastén, Manuel Silva, Joaquín Acosta, Macarena A. Varas, Miguel L. Allende, Andrés E. Marcoleta

**Affiliations:** 1 Laboratorio de Genómica Microbiana, Unidad Bioquímica, Departamento Biomédico, Facultad de Ciencias de la Salud, Universidad de Antofagasta, Antofagasta, Chile; 2 Millennium Institute Center for Genome Regulation, Facultad de Ciencias, Universidad de Chile, Santiago, Chile; 3 Grupo de Microbiología Integrativa, Facultad de Ciencias, Universidad de Chile, Santiago, Chile; 4 Vicerrectoría de Investigación y Postgrado, Universidad de La Serena, La Serena, Chile; 5 Facultad de Ciencias Forestales y de Conservación de la Naturaleza, Universidad de Chile, Santiago, Chile

**Keywords:** Atacama Desert, bioinnovation, cyanobacteria, extremophile microbial consortium, Nostocales, phycobilisome, phycocyanin, scytonemin

## Abstract

The Atacama Desert is one of the most extreme terrestrial environments on Earth, characterized by hyper-aridity, intense solar and ultraviolet radiation, oxidative stress, and metal-rich substrates. Here, we characterized an enriched cyanobacteria-associated consortium obtained from a high-altitude, river-influenced site in Quebrada Talabre, Atacama Desert. Deep shotgun metagenomic sequencing, achieving 99.7% estimated community coverage, enabled the reconstruction of 14 high-quality metagenome-assembled genomes representing five bacterial phyla. Genome-resolved analyses revealed multiple putative novel bacterial lineages, including a potential novel family-level lineage within Thermomicrobiales, putative novel species of *Allosphingosinicella*, *Flavobacterium*, and *Caulobacter*, and a distinct Nostocaceae lineage corresponding to the dominant cyanobacterium, tentatively named *Talabrenema galetoviciae* TAL-12. Functional annotation revealed marked differentiation among consortium members. Heterotrophic bacteria encoded predicted functions associated with aromatic compound degradation, nitrogen and sulfur turnover, iron acquisition, metal-biocide resistance, and oxidative-stress responses, whereas the TAL-12 cyanobacterial metagenome-assembled genome encoded the core machinery for oxygenic photosynthesis, carbon fixation, and pigment biosynthesis. Phylogenomic analyses supported *T. galetoviciae* TAL-12 as a novel species and likely novel genus within Nostocaceae. Comparative genomic and structural analyses revealed a diversified phycobiliprotein repertoire comprising phycocyanin, allophycocyanin, and phycoerythrocyanin components, together with divergent but structurally conserved paralogs and variable accessory pigment modules across related Nostocaceae genomes, consistent with enhanced light-harvesting flexibility. Pigment fractionation supported the absence of canonical phycoerythrin production and confirmed phycocyanin enrichment. Purified TAL-12 phycocyanin displayed moderate thermal stability, while the cyanobacterial component of TAL-12 consortium cultures retained chlorophyll during prolonged exposure to elevated temperature. In addition, the TAL-12 cyanobacterial genome encoded a Nostocales-like scytonemin biosynthetic gene cluster consistent with UV photoprotection, while lacking canonical cyanotoxin biosynthetic gene clusters. Together, these findings reveal previously underexplored bacterial diversity and predicted functional complementarity within an Atacama cyanobacteria-associated consortium, while identifying *T. galetoviciae* TAL-12 as a distinctive Nostocaceae lineage with light-harvesting, thermal-resilience, and photoprotective traits relevant to extremophile-based bioinnovation.

## Introduction

The Atacama Desert, located in northern Chile between latitudes 17°S and 27°S, is one of the most extreme terrestrial environments on Earth and is widely regarded as a natural laboratory for studying life under multiple environmental stressors (Azua-Bustos and González-Silva, 2014; Mörchen et al., 2019). Its hyper-arid core receives extremely low precipitation (0.5–12 mm year^-1^) (Tapia et al., 2018), resulting in minimal erosion rates and the accumulation of nitrates, halites, and metallic minerals in soils and sediments (Orellana et al., 2018; Schulze-Makuch et al., 2018). The characteristic climate of the Atacama Desert results from the combined effects of the Humboldt Current, persistent coastal winds, subtropical high-pressure systems, and the rain shadow imposed by the Andes Mountains. In addition to severe aridity, the region experiences exceptionally high solar irradiance associated with stratospheric ozone depletion (Cordero et al., 2016), with some of the highest annual UV-A and UV-B doses recorded worldwide, particularly in high-altitude inland areas (Cordero et al., 2018).

Despite these extreme conditions, the Atacama Desert hosts diverse and metabolically active microbial communities. Culture-independent approaches, including metabarcoding and metagenomic surveys of soils, lithic substrates, evaporitic deposits, and hypersaline environments, have revealed a remarkable diversity of bacteria, archaea, fungi, and microalgae inhabiting this region (Azua-Bustos et al., 2012; Crits-Christoph et al., 2016; Finstad et al., 2017). These microorganisms tolerate intense UV radiation, oxidative stress, desiccation, high salinity, temperature fluctuations, and toxic metals through a broad repertoire of adaptive strategies (Houston and Hartley, 2003; McKay et al., 2003; Orellana et al., 2018; Rampelotto, 2013). Such adaptations include the synthesis of specialized biomolecules and secondary metabolites, such as pigments, osmolytes, antioxidants, toxins, and exopolysaccharides, which contribute to cellular protection, biofilm formation, and environmental resilience (Raddadi et al., 2015) and have attracted growing interest for pharmaceutical, food, cosmetic, and biotechnological applications (Singh et al., 2016).

Among microorganisms from the Atacama Desert, cyanobacteria play a fundamental ecological role as primary producers, sustaining microbial ecosystems through oxygenic photosynthesis, carbon fixation, and, in many taxa, atmospheric nitrogen fixation (Briand et al., 2003; Mehdizadeh Allaf and Peerhossaini, 2022). These photoautotrophs are widely distributed across aquatic and terrestrial habitats, including deserts, polar regions, hypersaline systems, and geothermal environments, reflecting their remarkable physiological plasticity (Guedes et al., 2014). Within this phylum, members of the order Nostocales are particularly relevant in terrestrial and subaerial ecosystems due to their filamentous growth, the formation of heterocytes, specialized cells that support nitrogen fixation under appropriate conditions, extracellular matrix production, and ability to establish structured microbial assemblages such as mats, biofilms, and cyanosphere-associated consortia (Herrero et al., 2016; Hube et al., 2009; Saleem et al., 2025; Vilo et al., 2022).

Cyanobacterial consortia can comprise heterotrophic bacteria, archaea, fungi, and microalgae that collectively contribute to nutrient cycling, stress mitigation, and ecosystem stability. In microbial mats and cyanosphere-associated communities, cyanobacterial extracellular polymeric substances, oxygen gradients, and released organic compounds create structured microniches that support microbial interactions (Paerl and Pinckney, 1996; Stal, 2012). These associated microbiomes may enhance cyanobacterial fitness through complementary metabolic functions, detoxification processes, nutrient remineralization, and protection against environmental stressors, particularly under extreme conditions such as those found in the Atacama Desert (Hube et al., 2009; Vilo et al., 2022). Despite their ecological relevance, culturable cyanobacteria-associated microbial communities from the Atacama Desert remain poorly characterized, particularly at the genomic and functional levels.

A key adaptive feature underlying cyanobacterial success in extreme environments is their capacity for efficient light harvesting and chromatic acclimation under fluctuating and spectrally challenging light conditions. This capability is mediated by phycobiliproteins, accessory pigments that assemble into phycobilisomes, the major light-harvesting antenna complexes associated with the photosynthetic apparatus (Glazer, 1985; Grossman et al., 1993). By extending the range of usable wavelengths beyond those absorbed by chlorophyll, phycobiliproteins enhance photosynthetic efficiency under variable light regimes, including shaded, stratified, hypersaline, or high-irradiance environments (Kehoe and Gutu, 2006; Six et al., 2007). The principal phycobiliproteins include allophycocyanin (APC; 650–655 nm), phycocyanin (PC; 615–640 nm), phycoerythrin (PE; 565–575 nm), and, in some lineages, phycoerythrocyanin (PEC; 575–590 nm), which differ in spectral properties and spatial organization within the phycobilisome (Samsonoff and MacColl, 2001). In addition to their physiological roles in light harvesting and nitrogen storage, these pigments are widely explored as natural colorants, fluorescent probes, and bioactive compounds with antioxidant, anti-inflammatory, neuroprotective, and hepatoprotective properties (Cervantes-Llanos et al., 2018; Dahms et al., 2006; Galetovic et al., 2020; Sonani, 2016; Spolaore et al., 2006). Among them, phycocyanin is one of the most widespread and commercially relevant cyanobacterial pigments, and increasing attention has focused on phycobiliproteins from extremophilic cyanobacteria because harsh environments may select for enhanced pigment stability and distinctive biochemical properties (Dumay and Morançais, 2016; Fernández-Rojas et al., 2026; Glazer, 1994; Liu et al., 2022).

While phycobiliproteins optimize light harvesting, cyanobacteria inhabiting high-irradiance environments must also mitigate the damaging effects of excessive ultraviolet radiation. A major photoprotective strategy is the synthesis of scytonemin, a lipid-soluble indole-alkaloid pigment produced by several Nostocales and related cyanobacterial genera, including *Scytonema*, *Nostoc*, *Calothrix*, *Chlorogloeopsis*, and *Chroococcidiopsis* (Fuentes-Tristan et al., 2019; Garcia-Pichel and Castenholz, 1991). Scytonemin accumulates in the extracellular sheath or periplasm, where it functions as a passive UV-screening barrier capable of absorbing a substantial fraction of incident UV radiation. Its production, therefore, represents a key adaptive strategy enabling cyanobacteria to persist under intense solar irradiation, particularly in exposed terrestrial and desert environments (Gao and Garcia-Pichel, 2011; Gao et al., 2021; Garcia-Pichel and Castenholz, 1991; Soule et al., 2007). In several cyanobacteria, including *Nostoc punctiforme* and *Nodularia spumigena*, scytonemin biosynthesis is encoded by conserved gene clusters involved in pigment synthesis, regulation, and precursor supply pathways. However, their diversity, organization, and ecological significance remain incompletely understood across extremophilic cyanobacterial lineages (Soule et al., 2009; 2007).

Despite growing insights into cyanobacterial pigment biology and microbial diversity in the Atacama Desert, most studies in this region have relied predominantly on culture-independent approaches, leaving culturable cyanobacteria and their associated microbial communities comparatively underexplored. Consequently, little is known about the genome-resolved functional organization of cyanobacteria-associated communities inhabiting extreme desert environments, particularly in relation to light harvesting, stress adaptation, and photoprotection. Here, we characterized an enriched cyanobacteria-associated consortium obtained from a river-influenced site in Quebrada Talabre, a volcanic ravine within the Atacama Basin. For clarity, we use “TAL-12 consortium” to refer to the enriched microbial community characterized by shotgun metagenomics, whereas “*T. galetoviciae* TAL-12” refers specifically to its dominant Nostocaceae cyanobacterium. By integrating metagenomic reconstruction of the associated microbial community, comparative genomics, phylogenomics, pigment biochemistry, and protein characterization, we investigated the predicted functional differentiation among consortium members and performed an in-depth characterization of *Talabrenema galetoviciae* TAL-12. Together, these analyses provide insights into cyanobacteria-associated microbial systems from extreme desert environments and into the photobiological and biotechnological features of their dominant cyanobacterial member.

## Methods

### Consortium isolation, cultivation, and selective enrichment

Surface water-sediment slurry samples were collected during spring 2012 from the Talabre Ravine (23°18′56.1″S, 67°53′10.7″W), approximately 60 km from San Pedro de Atacama in the Atacama Salt Flat basin near the Láscar Volcano, at 3,300 m above sea level. Approximately 30 mL of this slurry was collected in sterile Falcon tubes and transported under refrigerated conditions (4 °C–8 °C) for up to 24 h before laboratory processing. After homogenization, 1 mL of the sediment-water slurry was inoculated into 20 mL of fresh nitrogen-free BG-11 (N−) medium. Cultures were maintained in liquid or solid BG-11 (N−) medium at 30 °C under continuous white fluorescent illumination (180 μE m^-2^ s^-1^), agitation (200 rpm), and aeration supplemented with 1% (v/v) CO_2_. Initial cyanobacterial growth was verified by light microscopy, after which cultures were transferred into fresh BG-11 (N−) medium. Growth was monitored through serial chlorophyll concentration measurements, and the exponential phase was identified from the log-linear increase in chlorophyll concentration, typically observed approximately 2 weeks after inoculation.

Exponentially growing TAL-12 consortium cultures were subjected to selective enrichment. A 15 mL aliquot was collected, and cyanobacterial filaments were recovered by centrifugation at 4,000 × g for 5 min at room temperature and washed with fresh medium. To reduce fast-growing heterotrophic bacteria, the resulting pellet was resuspended in 20 mL of BG-11 (N−) medium supplemented with gentamicin (1 mg mL^-1^), casamino acids (1.6 mg mL^-1^; Sigma-Aldrich), and D-glucose (0.8 mg mL^-1^; Sigma-Aldrich). Cultures were incubated for 48 h in darkness at 30 °C and 120 rpm using a benchtop incubator shaker (ZHWY-100B, ZHICHENG). Following incubation, cyanobacterial filaments were recovered by centrifugation, washed four times with fresh BG-11 (N−) medium, and regrown in liquid medium under standard culture conditions. Aliquots (200 µL) of the treated cultures were spread onto BG-11 (N−) agar plates supplemented with 3% (w/v) NaCl, corresponding to approximately 0.51 M, and individual filaments were transferred to fresh medium for further propagation. Biomass from exponentially growing cultures was collected by centrifugation (6,000 rpm, 15 min, 8 °C; Sorvall SS-34 rotor), washed four times with fresh medium, and used for total genomic DNA extraction.

### DNA extraction, metagenomic sequencing, and MAG reconstruction

Total genomic DNA was extracted from filament-enriched biomass pellets recovered from TAL-12 consortium cultures using the UltraClean Microbial DNA Isolation Kit (MoBio Laboratories, Inc.) according to the manufacturer’s instructions. DNA quality was assessed by electrophoresis on 0.8% agarose gels and quantified spectrophotometrically at 260 nm. Shotgun metagenomic libraries were prepared using the TruSeq DNA PCR-Free Library Kit with an average insert size of 350 bp and sequenced on an Illumina NovaSeq platform to generate 150-bp paired-end reads. Raw Illumina reads were quality-checked using FastQC v0.11.5, filtered with fastp v0.22.0 (Chen et al., 2018), and assembled using metaSPAdes v3.15.3 (Nurk et al., 2017). Taxonomic classification of metagenomic reads was performed using Kaiju v1.10.1 (Menzel et al., 2016) with the nr database, which assigns sequencing reads to taxa based on translated sequence similarity against a protein reference database. Metagenomic coverage was estimated using Nonpareil (Rodriguez-R et al., 2018b), a database-independent method based on sequence redundancy. Assembly quality was evaluated using QUAST v4.4 (Gurevich et al., 2013). Metagenome-assembled genomes (MAGs) were recovered through *de novo* binning using MetaBAT2 v2.17 (Kang et al., 2019), CONCOCT v1.1 (Alneberg et al., 2014), and MaxBin2 v2.2.4 (Wu et al., 2016). Resulting bins were combined and dereplicated using DAS Tool v1.1.2 (Sieber et al., 2018). MAG quality was assessed with CheckM (Parks et al., 2015), and bins with a quality score ≥50 (completeness −5 × contamination) were retained for downstream analyses.

General genome annotation was carried out using RASTtk v1.073 (Brettin et al., 2015), whereas functional and metabolic profiling were performed using DRAM v0.1.2 (Shaffer et al., 2020) and SUPER-FOCUS v1.4.1 (Silva et al., 2016). Metal and biocide resistance genes were identified using DIAMOND blastx (Buchfink et al., 2021) against the BacMet database of experimentally validated resistance determinants (Pal et al., 2014). Antibiotic resistance genes were predicted using RGI v6.0.5 and the CARD database v3.3.0 (Alcock et al., 2023).

### Taxonomic classification and phylogenomic analyses

Taxonomic assignment of the MAGs was performed using GTDB-Tk v2.3.2 (Chaumeil et al., 2022) with the GTDB R214 database. Maximum-likelihood phylogenetic trees were constructed using the multiple sequence alignment of 120 conserved single-copy bacterial marker proteins defined by the Genome Taxonomy Database and generated through the GTDB-Tk classify_wf pipeline. Phylogenetic reconstruction was conducted with RAxML v8.2.12 (Stamatakis, 2014) using the PROTGAMMAAUTO substitution model and 1,000 bootstrap iterations. Trees were visualized and formatted using iTOL v6.7.3 (Letunic and Bork, 2021). Average nucleotide identity (ANI) values between the TAL-12 cyanobacterial MAG and reference genomes were calculated using FastANI v1.32 (Jain et al., 2018). The closest publicly available genomes to the TAL-12 cyanobacterial MAG and the other recovered MAGs were further identified using the TypeMat, NCBI Prok, and MAGs databases implemented in MiGA v1.1.2.2 (Rodriguez-R et al., 2018a). The taxonomic placement of the TAL-12 cyanobacterial MAG relative to formally described taxa was additionally evaluated using the Type (Strain) Genome Server (TYGS) (Meier-Kolthoff et al., 2022).

### Biosynthetic gene cluster and cyanotoxin screening

The TAL-12 cyanobacterial MAG was analyzed using antiSMASH v7.1 (Blin et al., 2023) to identify biosynthetic gene clusters associated with secondary metabolism. In addition, targeted tBLASTn searches were performed against the nucleotide assembly of the TAL-12 cyanobacterial MAG with default parameters, an E-value threshold of 0.05, and a panel of 30 reference proteins from five characterized cyanotoxin biosynthetic pathways. The panel comprised the microcystin biosynthesis proteins McyA (AAF00960), McyB (AAF00961), McyC (AAF00962), McyD (AAF00959), McyE (AAF00958), and McyG (AAF00957); the nodularin biosynthesis proteins NdaA (AAO64403), NdaB (AAO64402), NdaC (AAO64404), NdaD (AAO64405), NdaE (AAO64406), and NdaF (AAO64407); the cylindrospermopsin biosynthesis proteins CyrA (ABX60160), CyrB (ABX60161), CyrC (ABX60163), CyrE (ABX60162), and CyrJ (ABX60159); the saxitoxin biosynthesis proteins SxtA (ABI75094), SxtB (ABI75093), SxtG (ABI75097), SxtI (ABI75099), SxtS (ABI75110), and SxtU (ABI75108); and the anatoxin-a/homoanatoxin-a biosynthesis proteins AnaA–AnaG (ACR33073–ACR33079). Returned alignments were evaluated according to amino acid identity, query coverage, E-value, and genomic location. Evidence for a canonical cyanotoxin biosynthetic gene cluster required the recovery and genomic colocalization of multiple pathway-specific components in an organization compatible with a characterized cluster. Isolated matches to conserved enzymatic domains, particularly NRPS and PKS domains, were not considered sufficient evidence of a cyanotoxin biosynthetic pathway.

### Comparative analysis of the scytonemin biosynthetic gene cluster

Genomic regions encompassing the scytonemin biosynthetic gene cluster were extracted from the annotated TAL-12 cyanobacterial MAG and from representative annotated Nostocales genomes. The resulting regions were compared using clinker (Gilchrist and Chooi, 2021), which was also used to generate the comparative gene cluster visualization. Pairwise sequence similarities among homologous genes were represented in the resulting cluster comparison.

### Targeted amplification and nanopore sequencing of phycocyanin genes

Genomic DNA from TAL-12 consortium cultures was used for targeted amplification of regions encoding the alpha (PCα) and beta (PCβ) subunits of phycocyanin (PC). The PCβ region was amplified using primers PCβ-F (5′-GGC​TGC​TTG​TTT​ACG​CGA​CA-3′) and PCβ-R (5′-CCA​GTA​CCA​CCA​GCA​ACT​AA-3′), whereas the PCα region was amplified using primers PhycoA_F (5′-ATGAAAACNCCKWTWACTGAAGC-3′) and PhycoA_R (5′-TTAGCTGAGGGCRTTRATGGCGTA-3′). PCR amplifications were performed in a final reaction volume of 25 µL. The amplification program consisted of an initial denaturation at 95 °C for 4 min, followed by 35 cycles of denaturation at 95 °C for 1 min, annealing at 58 °C for 20 s, and extension at 72 °C for 1 min, with a final extension at 72 °C for 5 min and subsequent holding at 4 °C. The expected amplicon sizes were 486 bp for PCα and 286 bp for PCβ. Amplified products were visualized by electrophoresis on 0.8% agarose gels and purified using the Wizard SV Gel and PCR Clean-Up System (Promega), following the manufacturer’s instructions. Purified amplicons were sequenced using a MinION Mk1C device with an R9 flow cell and the Oxford Nanopore Ligation Sequencing Kit SQK-LSK109 with native barcoding. Sequencing was performed for 3 h. Basecalling and sequence quality assessment were conducted using Guppy v3.1.5. Consensus sequences for the PCα and PCβ amplicons were generated from the Nanopore reads using the Oxford Nanopore EPI2ME wf-amplicon workflow.

### Phylogenetic analyses of phycobiliproteins and ScyB

Phylogenetic analyses were performed using the PCα amino acid sequence translated from the Nanopore consensus nucleotide sequence obtained for the *T. galetoviciae* TAL-12 PCα amplicon and the ScyB amino acid sequence identified in the TAL-12 cyanobacterial MAG. Reference sequences, accession numbers, and source organisms are listed in Supplementary Table S1. Multiple sequence alignments were generated using the MUSCLE algorithm (Edgar, 2022) implemented in MEGA v11.0.10 (Tamura et al., 2021). Phylogenetic trees were reconstructed using the Neighbor-Joining method in MEGA with 5,000 bootstrap replicates. Conserved amino acid regions were examined using GeneDoc v2.7. For the ScyB analysis, ambiguously aligned positions were removed using Gblocks v0.91b with default settings, yielding a final alignment of 342 amino acid positions. The ScyB phylogenetic tree was reconstructed from this filtered alignment.

### Comparative profiling of phycobiliprotein genes

The distribution of phycobiliprotein genes across representative Nostocaceae genomes was assessed using tBLASTn searches with reference phycobiliprotein sequences as queries against each genome. Hits were retained when they showed at least 50% amino acid identity and 80% query coverage. These thresholds were selected to reduce potential false-positive assignments while allowing for sequence divergence among the different Nostocaceae taxa included in the analysis. In addition to sequence similarity, the genomic context of each candidate gene was examined using the corresponding genome annotations to support gene identification and distinguish among related variants and paralogs. Gene identity and variant assignment were therefore based on the combined evidence of sequence similarity and characteristic genomic neighborhood. The resulting presence or absence profiles were integrated with the phylogenomic analysis to compare the distribution of phycocyanin, allophycocyanin, phycoerythrocyanin, and phycoerythrin genes across Nostocaceae genomes.

### Structural prediction and comparison of phycobiliproteins

Three-dimensional structures of *T. galetoviciae* TAL-12 phycobiliproteins were predicted using AlphaFold2 v2.3.2 (Jumper et al., 2021). Predicted structures were compared by structural superposition using US-align v20241108 (Zhang et al., 2022). Superimposed structural models were subsequently visualized and rendered using PyMOL v3.1.0 (Schrödinger, LLC). These analyses were used to assess structural conservation among the divergent phycobiliprotein subunits identified in the TAL-12 cyanobacterial MAG.

### Phycocyanin purification

Phycocyanin (PC) purification was performed according to a previously described method with minor modifications (Patel et al., 2005). Cyanobacterial biomass recovered from TAL-12 consortium cultures was resuspended in 50 mM phosphate buffer (pH 7.0) containing lysozyme (1.0 mg mL^-1^ final concentration) and incubated at 37 °C for 1 h. Cells were subsequently disrupted by sonication in an ice bath for 2 min using 15-s intervals. The crude extract was diluted 1:1 with phosphate buffer and centrifuged at 12,000 × g for 15 min to remove cell debris. Selective precipitation of phycobiliproteins was carried out by gradual ammonium sulfate addition under continuous stirring at 4 °C. Saturation ranges of 0%–35% and 35%–60% were used to enrich phycoerythrin- and phycocyanin-containing fractions, respectively. Following overnight incubation, protein precipitates were recovered by centrifugation at 12,000 rpm for 15 min and resuspended in phosphate buffer. Samples were dialyzed overnight at 4 °C using 12 kDa molecular weight cut-off cellulose dialysis tubing (Sigma-Aldrich). Further purification of PC was performed by ion-exchange chromatography using a DEAE-cellulose column and elution with a 0.03–1.0 M NaCl gradient at room temperature. Blue-colored fractions were pooled, and UV-visible absorption spectra were recorded using a Jasco V-730 spectrophotometer. Purified PC fractions were subsequently lyophilized and stored at −20 °C until further analyses.

### Phycocyanin thermal stability assays

Purified PC solutions (50 μg mL^-1^) prepared in 50 mM phosphate buffer (pH 7.0) were incubated in triplicate at temperatures ranging from 4 °C to 80 °C for 72 h. Visible absorption spectra (400–700 nm) were recorded every 24 h to evaluate thermal stability. PC concentration was determined spectrophotometrically according to Tandeau de Marsac and Houmard (1988), using the equation: [PC] (mg mL^-1^) = (A620–0.7 × A650)/7.38. In this equation, the term 0.7 × A650 corrects the absorbance at 620 nm for the contribution of allophycocyanin, whereas 7.38 corresponds to the specific absorption coefficient used in the original method to convert the corrected absorbance into PC concentration.

### Thermal exposure assays of TAL-12 consortium cultures

TAL-12 consortium cultures were incubated in triplicate at 50 °C for 15 days to evaluate the response of the cyanobacterial component to prolonged heat exposure. Control cultures were maintained at 22 °C under standard growth conditions. Samples were collected after 0, 1, 2, 3, 4, and 15 days of incubation. Biomass was recovered by centrifugation at 10,000 rpm for 5 min, resuspended in pure acetone, and incubated overnight at 4 °C in darkness for chlorophyll extraction. Chlorophyll concentration in acetone extracts was determined spectrophotometrically at 663 nm using an extinction coefficient of 92.6 g^-1^ L cm^-1^ (Brenowitz and Castenholz, 1997).

## Results

### An enriched cyanobacteria-associated consortium from the Atacama Desert harbors multiple putative novel bacterial lineages

To investigate microbial adaptation and potential biotechnological traits associated with extreme desert environments, we analyzed an enriched cyanobacteria-associated consortium established from a surface water-sediment slurry collected in the Talabre Ravine, Atacama Desert. This ravine is a high-altitude, river-influenced system located near 3,300 m above sea level, originating on the slopes of the active Láscar Volcano and draining toward the Atacama Salt Flat basin. The sampling site, therefore, represents a hydrological corridor embedded within a hyper-arid landscape exposed to intense solar and ultraviolet radiation. Although direct UV measurements are not available for Talabre Ravine, UV-B irradiance values up to ∼5.5 W m^-2^ have been reported for nearby high-altitude Atacama sites such as Llano de Chajnantor, supporting the inference that Talabre is also subjected to strong UV exposure, potentially amplified by light-colored volcanic substrates.

During culture establishment, the original sediment-derived community was subjected to successive selective steps, including growth under nitrogen-limiting conditions, repeated washing of cyanobacterial filaments, and antibiotic treatment intended to reduce fast-growing heterotrophic bacteria. Following these procedures, an enriched microbial community remained associated with the cyanobacterial filaments and could be reproducibly maintained under laboratory conditions. The present metagenomic analysis therefore characterizes the consortium recovered after selective enrichment. Because community composition was not assessed after each enrichment step, persistence of the established culture should not be interpreted as evidence of compositional stability throughout the enrichment process.

To resolve the composition and genomic potential of this consortium, we applied deep shotgun metagenomic sequencing. Approximately 16 Gbp of Illumina sequencing data were generated from total community DNA. Nonpareil, a database-independent method that estimates metagenomic coverage based on sequence redundancy (Rodriguez-R et al., 2018b), estimated 99.7% coverage, indicating that the sequencing effort captured most of the abundance-weighted sequence diversity present in the consortium (Supplementary Figure S2A). Taxonomic profiling of the metagenomic reads showed that nearly 50% of the sequences were assigned to Cyanobacteriota, predominantly within the order Nostocales (Supplementary Figure S2B). Pseudomonadota represented the second most abundant phylum, accounting for 42% of the reads and mainly comprising Alphaproteobacteria affiliated with Sphingomonadales, Caulobacterales, and Rhodobacterales, together with members of Burkholderiales. Additional taxa included Actinomycetota (4.7%), primarily represented by Propionibacteriales and Pseudonocardiales, and Bacteroidota (4.4%), predominantly associated with Flavobacteriales. Overall, the TAL-12 consortium comprised representatives from at least five bacterial phyla and multiple phylogenetically distinct lineages, with cyanobacteria as the dominant community component.

Genome-resolved metagenomic reconstruction using an integrated binning and dereplication strategy yielded fourteen high-quality metagenome-assembled genomes (MAGs). The recovered MAGs showed completeness values ranging from 92.69% to 100% and contamination levels between 0% and 2.9% (Table 1). These genomes were assigned to five bacterial phyla: Pseudomonadota (9 MAGs), Actinomycetota (2 MAGs), Cyanobacteriota (1 MAG), Bacteroidota (1 MAG), and Chloroflexota (1 MAG). Genome-based taxonomic classification with GTDB-Tk revealed several lineages with substantial taxonomic novelty. This inference was supported by the high quality of the reconstructed genomes, together with their genetic divergence from currently represented taxa in GTDB. Particularly notable cases included a Chloroflexota MAG assigned only at the order level within Thermomicrobiales, suggesting a putative novel family-level lineage; a Bacteroidota MAG corresponding to a putative novel species within the genus *Flavobacterium*; and two Pseudomonadota MAGs representing putative novel species within the genera *Allosphingosinicella* and *Caulobacter*. In addition, the cyanobacterial MAG was assigned to the family Nostocaceae but not confidently placed within any described genus, consistent with a putative novel genus-level lineage within Nostocales.

**TABLE 1:** Main features of the metagenome-assembled genomes reconstructed from the enriched TAL-12 consortium.

MAG	Phylum	GTDB taxonomy	Complete-ness (%)	Contami-nation (%)	Assembly length (bp)	N° contigs	N50 (bp)
1	Actinomycetota	*Pseudonocardia alni*	92.69	2.9	5,781,784	591	13,021
2	Pseudomonadota	*Allosphingosinicella* sp.	100.0	0.87	3,529,319	34	170,519
3	Bacteroidota	*Flavobacterium* sp.	99.29	0.35	4,203,803	10	662,556
4	Pseudomonadota	*Comamonas tsuruhatensis*	100.0	0.46	6,489,157	68	216,786
5	Chloroflexota	o_Thermomicrobiales	100.0	0.94	5,534,929	224	488,245
6	Actinomycetota	*Nocardioides* sp013155335	99.48	0.65	4,755,766	55	185,953
7	Pseudomonadota	*Stenotrophomonas maltophilia*	96.72	0.0	4,570,597	127	57,640
8	Pseudomonadota	*Herbaspirillum huttiense*	99.87	0.0	5,679,930	31	339,807
9	Cyanobacteriota	f_Nostocaceae	99.78	0.33	5,704,466	44	215,657
10	Pseudomonadota	*Caulobacter* sp.	99.51	0.67	5,631,752	55	161,630
11	Pseudomonadota	*Brevundimonas aurantiaca*	100.0	0.0	3,428,501	18	612,061
12	Pseudomonadota	*Tardiphaga* sp.	94.4	2.38	5,777,303	651	12,243
13	Pseudomonadota	*Sphingomonas zeae*	96.53	2.45	3,423,935	90	59,106
14	Pseudomonadota	*Paracoccus marcusii*	99.39	1.31	3,629,534	22	229,452

Together, these results show that the enriched TAL-12 consortium comprises representatives of at least five bacterial phyla and harbors several putatively novel bacterial lineages. The near-complete metagenomic coverage and recovery of 14 high-quality MAGs provide a genome-resolved framework for examining predicted functional differentiation among consortium members.

### Genome-resolved profiling reveals predicted functional differentiation among TAL-12 consortium members

We compared the predicted functional repertoires encoded by the 14 reconstructed MAGs using SUPER-FOCUS. Central carbohydrate metabolism, protein biosynthesis and degradation, and other core cellular functions were broadly represented across the recovered genomes (Figure 1). To identify potentially differentiating traits, subsequent analyses focused on predicted pathways that were unevenly distributed among consortium members and were relevant to organic compound turnover, nitrogen and sulfur transformations, iron acquisition, cofactor metabolism, and phototrophy.

**FIGURE 1:**
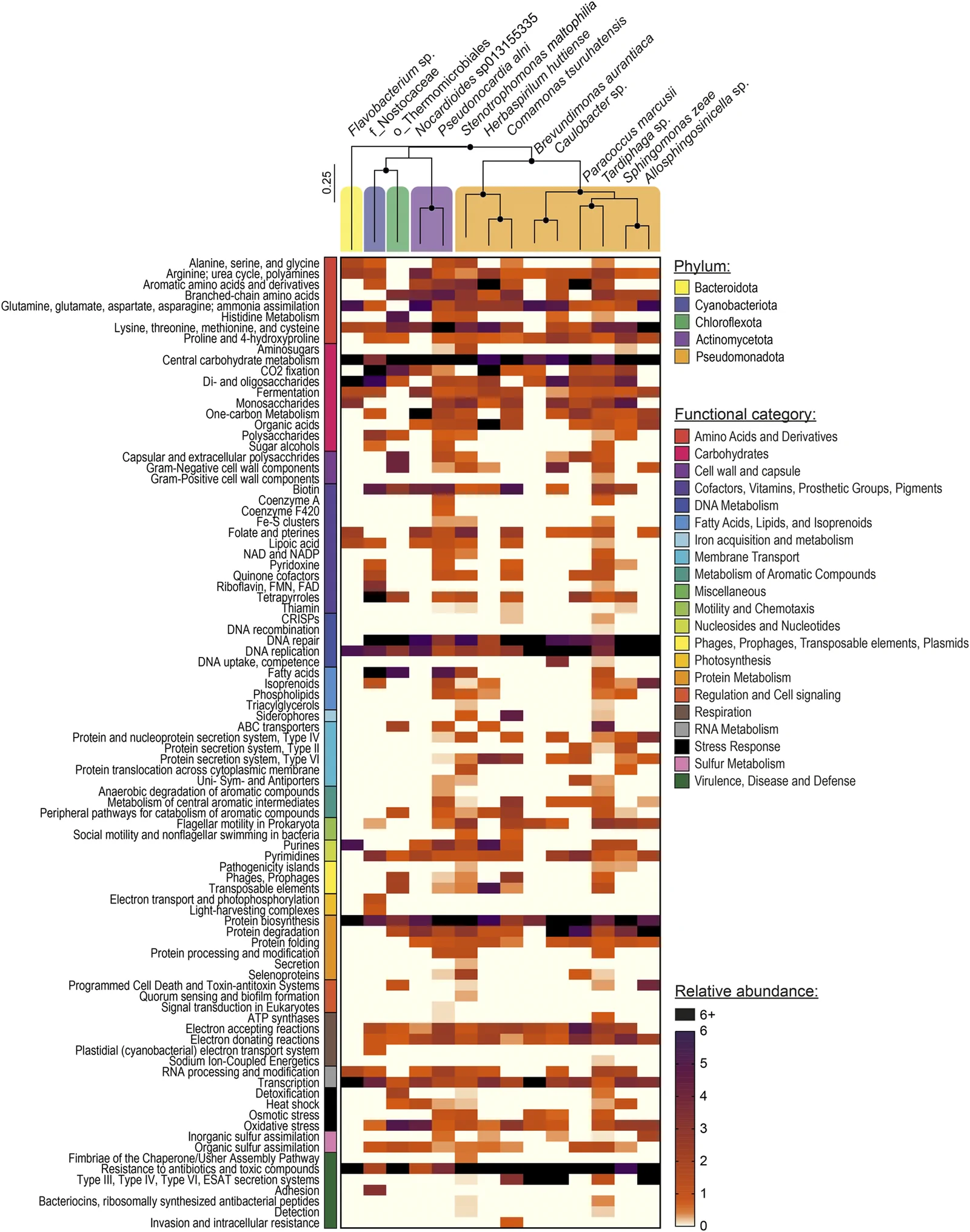
Functional profile of reconstructed genomes from the TAL-12 consortium. Heatmap showing the relative abundance of functional categories annotated in each metagenome-assembled genome (MAG) using SUPER-FOCUS. Functional categories are grouped according to SEED subsystems and displayed for each MAG ordered by taxonomic affiliation. Color intensity represents the relative abundance of genes assigned to each functional category within each genome. Shared functional categories and lineage-specific enrichments illustrate differences in the predicted functional repertoires of consortium members.

Genes associated with aromatic compound degradation were concentrated primarily in the *Pseudonocardia*, *Tardiphaga*, and *Comamonas* MAGs. *Pseudonocardia* encoded the broadest predicted repertoire, including functions associated with the degradation of hydroxyaromatic, N-heterocyclic, benzoate-related, salicylate, gentisate, and p-hydroxybenzoate compounds. It was also the only MAG in which the meta-cleavage pathway was detected. *Tardiphaga* encoded predicted pathways for aromatic amine, benzoate, and gentisate degradation and was the only MAG containing genes associated with anaerobic benzoate metabolism. The *Comamonas* MAG also encoded a broad repertoire of aromatic catabolic functions, with particularly strong representation of genes associated with salicylate and gentisate degradation.

Predicted functions related to polysaccharide and nutrient turnover were also unevenly distributed. The *Stenotrophomonas* MAG encoded genes associated with chitin, N-acetylglucosamine, N-acetylgalactosamine, and galactosamine utilization and was the only MAG with predicted alginate metabolism. The *Flavobacterium* MAG contained genes associated with trehalose and other di- and oligosaccharide utilization, whereas the *Caulobacter* MAG was enriched in genes encoding aminopeptidases, serine endopeptidases, and other protein-degradation functions.

Genes related to nitrogen and sulfur transformations showed additional lineage-specific patterns. The *Pseudonocardia* MAG encoded predicted functions associated with allantoin and cyanate utilization, ammonia assimilation, dissimilatory nitrite reduction, and nitrate and nitrite ammonification. The *Herbaspirillum* MAG contained genes associated with urea decomposition, whereas the *Allosphingosinicella* MAG showed the strongest representation of genes involved in inorganic sulfur assimilation. Predicted iron-acquisition functions, including siderophore-related genes, were detected principally in *Tardiphaga*, *Stenotrophomonas*, and *Comamonas*, with the latter encoding genes associated with pyoverdine biosynthesis. Several of these heterotrophic MAGs also encoded comparatively broad repertoires of genes associated with cofactor, quinone, carotenoid, and isoprenoid biosynthesis.

In contrast, the TAL-12 cyanobacterial MAG was distinguished by the predicted genetic machinery for oxygenic photosynthesis, CO_2_ fixation, β-carboxysome formation, photosynthetic electron transport, photophosphorylation, light harvesting, and phycobilisome assembly. It also encoded genes associated with tetrapyrrole and chlorophyll metabolism, carotenoid biosynthesis, and fatty acid and lipid metabolism, including polyunsaturated fatty acid biosynthesis.

Overall, genome-resolved annotation revealed marked differentiation in the predicted functional repertoires of consortium members. The TAL-12 cyanobacterial MAG encoded the principal functions associated with phototrophic primary production, whereas heterotrophic MAGs contained differentiated predicted repertoires related to aromatic compound degradation, polysaccharide and nutrient turnover, nitrogen and sulfur transformations, iron acquisition, and cofactor metabolism. This distribution is consistent with potential functional complementarity within the enriched consortium, although metabolic exchange and functional interactions among its members were not directly measured.

### Predicted stress-response and resistance functions are unevenly distributed across the TAL-12 consortium

Given the extreme environmental conditions of the Atacama Desert, we examined the distribution of predicted functions associated with stress response and resistance to toxic compounds across the TAL-12 consortium. SUPER-FOCUS annotation identified genes associated with oxidative stress, heat shock, osmotic stress, and responses to chemically challenging compounds (Supplementary Figure S3). These functions were unevenly distributed among the reconstructed genomes. The Stenotrophomonas, Tardiphaga, Comamonas, Herbaspirillum, and *Caulobacter* MAGs showed the broadest predicted stress-response repertoires, including genes associated with hydrogen peroxide detoxification, superoxide dismutation, and responses to detergents, solvents, phenolic compounds, quaternary ammonium compounds, and alkanes. In contrast, the *Flavobacterium*, Allosphingosinicella, and TAL-12 cyanobacterial MAGs contained fewer annotated stress-associated functions.

Targeted searches against the BacMet database revealed additional differences in the distribution of putative metal and biocide resistance determinants (Figure 2). Genes associated with resistance or homeostasis involving arsenic, cadmium, chromium, cobalt, copper, mercury, nickel, zinc, and iron were detected across multiple MAGs, together with genes associated with responses to organic biocides and oxidative stressors. The broadest repertoires were again observed in Stenotrophomonas, Tardiphaga, Comamonas, Herbaspirillum, and *Caulobacter* and included predicted efflux systems, metal-binding proteins, redox-transforming enzymes, and detoxification functions.

**FIGURE 2:**
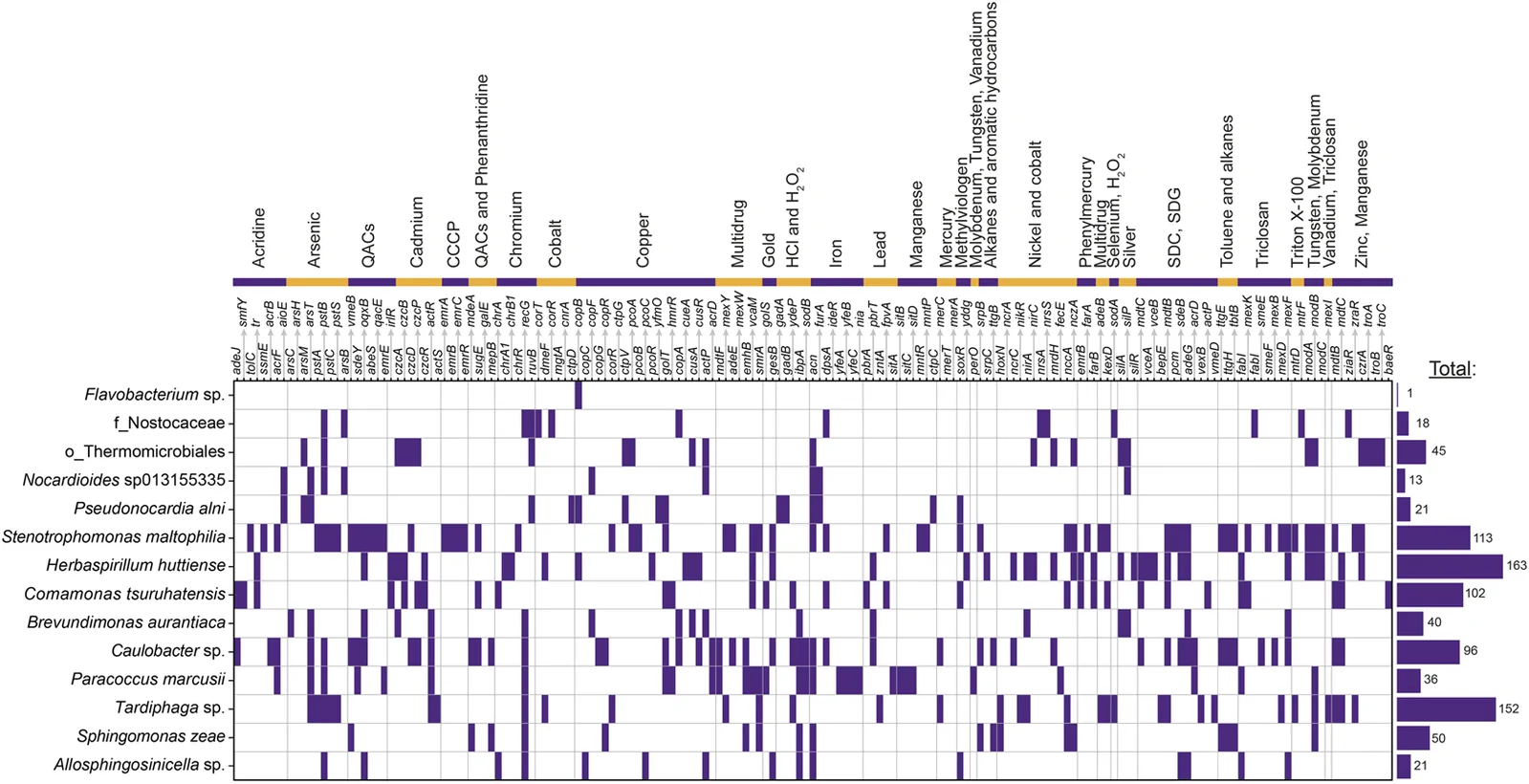
Distribution of predicted metal and biocide resistance determinants across TAL-12 consortium members. The heatmap summarizes the presence and relative abundance of genes associated with resistance or homeostasis involving toxic metals, biocides, and chemical stressors, identified through sequence similarity searches against the BacMet database. Predicted determinants are grouped by target compound or stressor and displayed for each MAG. The bar plot shows the total number of annotated resistance-associated genes per genome. Their uneven distribution illustrates lineage-specific differences in predicted resistance repertoires.

Putative mercury-resistance components, including homologs of *merA* and *merR*, were detected principally in the *Tardiphaga*, *Stenotrophomonas*, *Comamonas*, and *Herbaspirillum* MAGs. Genes associated with resistance to phenolic compounds, quaternary ammonium compounds, detergents, alkanes, aromatic hydrocarbons, and other organic biocides were particularly represented in Stenotrophomonas and *Comamonas*. The TAL-12 cyanobacterial MAG showed a more restricted predicted resistance repertoire, primarily comprising functions associated with iron and copper homeostasis and oxidative-stress responses.

Collectively, these analyses reveal lineage-specific differences in the predicted stress-response and resistance repertoires encoded by TAL-12 consortium members. Their uneven distribution is consistent with potential functional differentiation among the recovered genomes but does not, by itself, demonstrate community-level stress resilience or functional interactions among consortium members.

### Phylogenomics supports *Talabrenema galetoviciae* TAL-12 as a novel Nostocaceae lineage

Genome-based taxonomic classification using GTDB-Tk placed the TAL-12 cyanobacterial MAG within the family Nostocaceae, close to representatives assigned to the genus *Nodularia*. The closest genome available in GTDB corresponded to the uncharacterized species sp015207155. The TAL-12 cyanobacterial MAG shared an average nucleotide identity (ANI) of 93.94% with this genome, below the commonly accepted 95% threshold for species-level circumscription and consistent with its classification as a distinct species.

As an independent classification approach, we used the Microbial Genome Atlas Online (MiGA) TypeMat tool to identify the closest genomes from formally named taxa and assess taxonomic novelty. MiGA assigned the TAL-12 cyanobacterial MAG most likely to the order Nostocales (p = 0.0024), probably to the family Nostocaceae (p = 0.028), and possibly to the genus *Dendronalium* (p = 0.24). MiGA also indicated that the MAG represents a species not included in the TypeMat database (p = 0.0025). Consistently, analysis with the Type Strain Genome Server supported its placement as a novel species not represented in the TYGS database, with the type strains of *Athlanticothrix* silvestris and *Halotia branconii* identified as its closest formally described relatives.

To resolve the inconsistent genus-level assignments obtained with database-based tools, we performed a phylogenomic analysis using representative Nostocales genomes available in GTDB. The analysis was based on a concatenated alignment of 120 conserved single-copy marker proteins defined by GTDB (Figure 3). In the resulting maximum-likelihood tree, the TAL-

**FIGURE 3:**
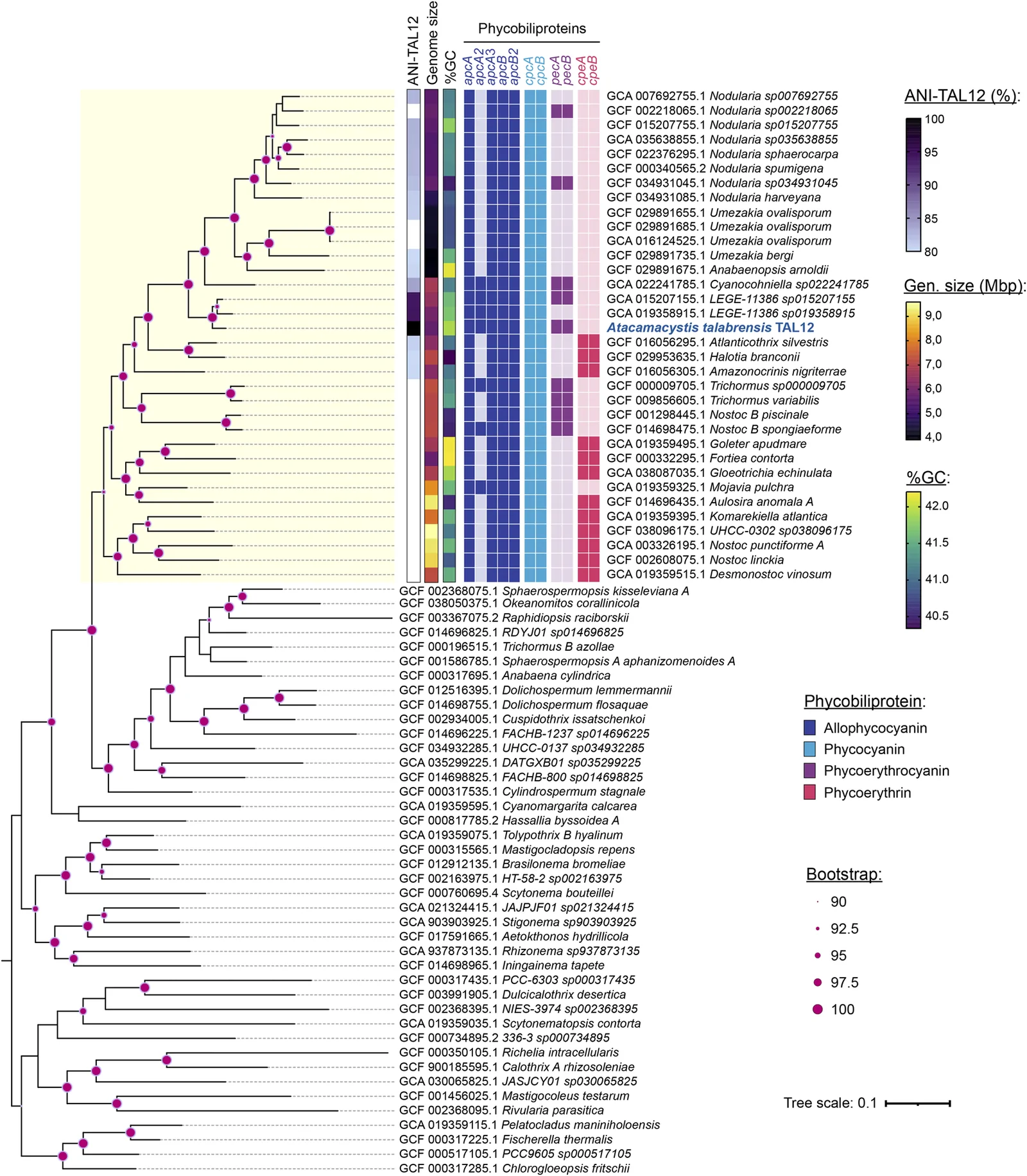
Phylogenomic placement of TAL-12 within Nostocales and comparative distribution of phycobiliprotein genes. Maximum-likelihood phylogenomic tree based on a concatenated alignment of 120 conserved single-copy marker proteins defined by the Genome Taxonomy Database (GTDB). The tree includes the TAL-12 cyanobacterial MAG and representative Nostocales genomes. Bootstrap support values ≥ 70% are indicated at nodes. TAL-12 forms a distinct and well-supported lineage within Nostocaceae, separated from currently described genera and positioned near *Nodularia*, *Dendronalium*, *Athlanticothrix*, *Halotia*, *Cyanocohniella*, and LEGE 11386-related genomes. The adjacent heatmap summarizes ANI values relative to TAL-12, genome size, GC content, and presence/absence of selected phycobiliprotein genes, colored by pigment class. Core allophycocyanin and phycocyanin genes are broadly conserved, whereas *apcA2*, *pecAB*, and *cpeAB* display more restricted distributions, suggesting diversification of accessory light-harvesting modules across Nostocales.

12 cyanobacterial MAG formed a distinct and well-supported lineage within Nostocaceae. Although positioned near lineages assigned to *Nodularia*, *Dendronalium*, *Athlanticothrix*, *Halotia*, and *Cyanocohniella*, it did not cluster within any of these genera. Instead, it formed an independent branch with a phylogenetic separation comparable to that observed among recognized Nostocaceae genera. This topology supports the classification of *T. galetoviciae* TAL-12 as a novel species and is consistent with a putative novel genus-level lineage within Nostocaceae.

Among its closest phylogenomic neighbors was GCA_015207155.1, corresponding to Nostocales cyanobacterium LEGE 11386, an isolate obtained from an intertidal marine sponge in Portugal. This genome is 6,494,076 bp in length and has a GC content of 41.61%. Another related genome, GCA_022241785.1, corresponds to *Cyanocohniella* sp. LLY, reconstructed from the edible cyanobacterial macrocolony “Llayta” collected in 2014 from Visviri in northern Chile. This genome comprises 6,781,030 bp and has a GC content of 41.17% (Vilo et al., 2022). In comparison, the *T. galetoviciae* TAL-12 genome is smaller, at 5,704,466 bp, while exhibiting a similar GC content of 41.91%. Despite occupying a related phylogenomic neighborhood, their genomic divergence, branch separation, and ANI values support the recognition of *T. galetoviciae* TAL-12 as a distinct Nostocaceae lineage.

### Expanded phycobilisome gene repertoire suggests chromatic flexibility in *Talabrenema galetoviciae* TAL-12

Genome analysis showed that *T. galetoviciae* TAL-12 encodes the core genetic machinery for oxygenic photosynthesis, including components of photosystems I and II, photosynthetic electron transport, and carbon fixation. The TAL-12 cyanobacterial MAG also encoded a diversified phycobilisome-associated repertoire that likely constitutes its principal light-harvesting antenna system. Because phycobilisome composition influences the spectral range of light harvesting, we examined the organization and diversity of these genes to assess the predicted light-harvesting flexibility of *T. galetoviciae* TAL-12.

Detailed inspection of the *T. galetoviciae* TAL-12 genome revealed a diversified repertoire of phycobilisome-associated proteins distributed across multiple genomic regions (Figure 4). In total, nine genes encoding phycobiliprotein subunits were identified, corresponding to phycocyanin (PC), phycoerythrocyanin (PEC), and allophycocyanin (APC) components. A primary phycocyanin/phycoerythrocyanin region encoded C-phycocyanin α- and β-subunits (*cpcA* and *cpcB*), multiple phycobilisome rod-core linker polypeptides (*cpcG*), rod-associated linker proteins (*cpcC* and *cpcD*), and phycocyanobilin lyases (*cpcE*/*cpcF*). Immediately downstream, this region extended into a phycoerythrocyanin module containing *pecA*, *pecB*, *pecC*, *pecE*, and *pecF*, suggesting genomic linkage between PC- and PEC-associated light-harvesting modules. Notably, this PC/PEC region was located close to genes encoding photosystem components, including photosystem II genes such as *psbB*, *psbT*, *psbA*, *psbD*, and *psbC*, as well as photosystem I-associated genes such as *ycf4* and *psaC*. This organization places the phycobilisome gene repertoire within a broader genomic context associated with photosynthetic electron transport.

**FIGURE 4:**
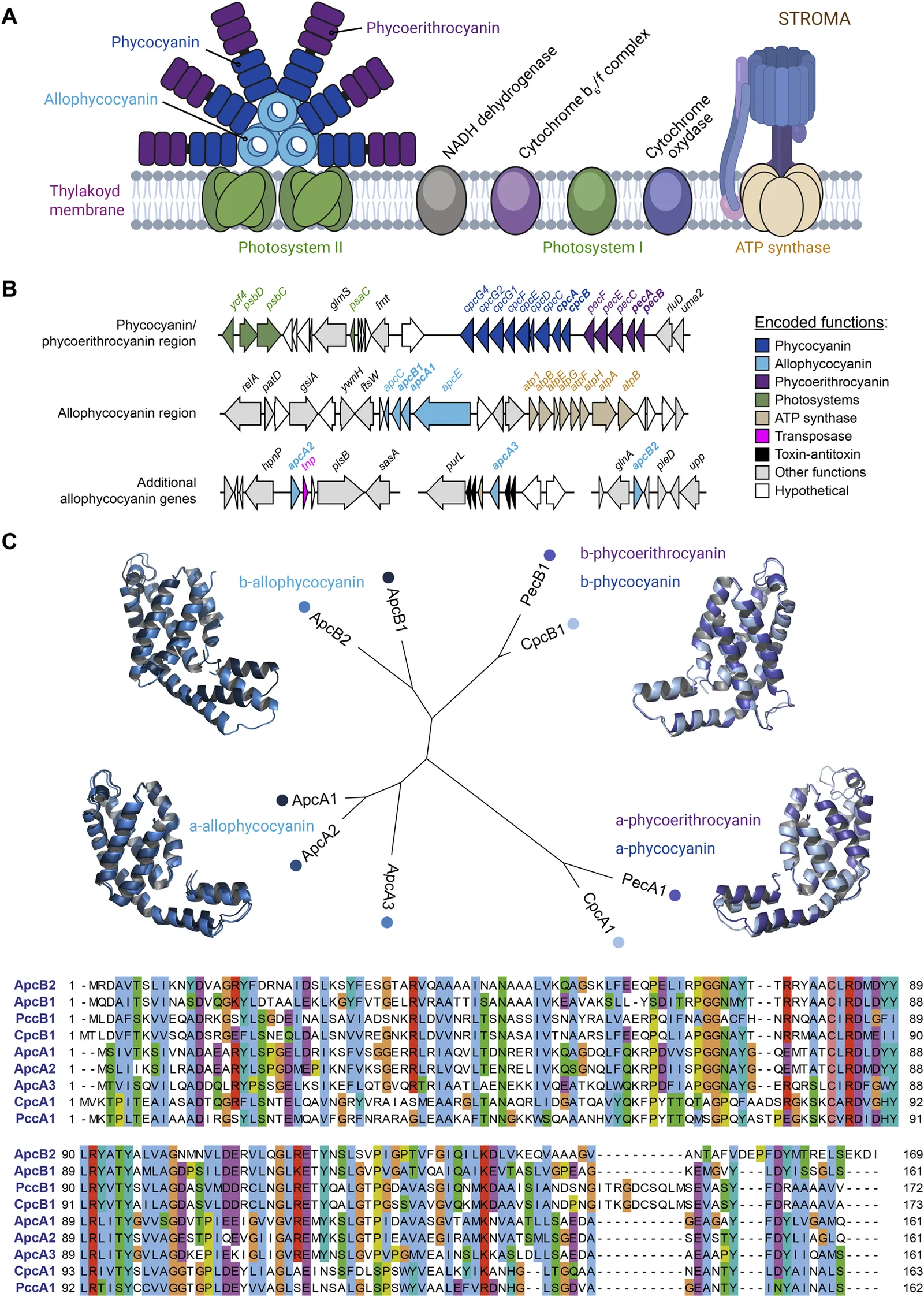
Photosynthetic architecture and phycobiliprotein repertoire of *Talabrenema galetoviciae* TAL-12. **(A)** Schematic representation of the photosynthetic apparatus encoded by *T. galetoviciae* TAL-12, showing photosystem II, photosystem I, electron transport components, ATP synthase, and associated phycobilisome antenna complexes. Phycobiliproteins identified in TAL-12 include phycocyanin, allophycocyanin, and phycoerythrocyanin, suggesting an expanded antenna system potentially suited to variable spectral conditions. **(B)** Genomic organization of phycobiliprotein-associated regions in the TAL-12 genome. A main phycocyanin/phycoerythrocyanin region contains *cpc* and *pec* genes encoding phycobiliprotein subunits, linker proteins, and bilin-associated enzymes, located near photosystem-related genes. A separate allophycocyanin region contains canonical *apcA1* and *apcB1* genes together with additional APC-associated components and occurs near ATP synthase genes. Additional isolated APC genes, including *apcA2*, *apcA3*, and *apcB2*, are in distant genomic regions with no obvious photosynthesis-related neighboring genes. The proximity of *apcA2* to a transposase and the association of *apcA3* with putative toxin-antitoxin genes suggest a dynamic evolutionary history potentially involving duplication, horizontal transfer, or genome rearrangement. **(C)** Comparative analysis of the nine phycobiliproteins encoded by TAL-12. The central tree summarizes sequence divergence among phycocyanin, phycoerythrocyanin, and allophycocyanin subunits. Predicted structural alignments and multiple sequence comparisons reveal conservation of the characteristic phycobiliprotein fold and key residues despite sequence variation, suggesting that the additional paralogs may remain functional and contribute to expanded chromatic or light-harvesting diversity.

The allophycocyanin-associated genes showed a distinct genomic organization. Canonical *apcA1* and *apcB1* genes encoding APC α- and β-subunits were found in a separate genomic region together with additional APC-related genes, including *apcE* and linker-associated components. This APC region occurred near genes encoding ATP synthase subunits, reinforcing the physical separation between PC/PEC rod-associated modules and APC core-associated components. In contrast, additional putative APC genes, including *apcA2*, *apcA3*, and *apcB2*, were found isolated in distant genomic regions lacking obvious photosynthesis- or phycobilisome-related neighboring genes. The presence of a transposase downstream of *apcA2*, together with putative toxin-antitoxin genes flanking *apcA3*, suggests that at least some of these additional APC copies may have been shaped by horizontal gene transfer, gene duplication, or genome rearrangement. The isolated *apcB2* copy showed a similar dispersed organization, although no nearby mobility-associated genes were detected in this case.

The co-occurrence of PC, APC, and PEC in *T. galetoviciae* TAL-12 is consistent with a potentially broadened light-harvesting repertoire. PEC is less widely distributed than PC and APC and has been associated with green-light utilization in shaded or spectrally filtered environments. Comparative sequence and structural analyses provided additional evidence that the nine identified phycobiliprotein subunits retain the characteristic phycobiliprotein fold and conserved residues expected for their respective subunit classes (Figure 4C). Despite substantial sequence divergence, the predicted structures were strongly conserved. These results are consistent with structurally intact phycobiliprotein variants but do not demonstrate their expression, assembly into functional phycobilisomes, or individual spectral properties.

Together, the multiplicity, diversity, structural conservation, and genomic dispersion of phycobiliprotein genes in *T. galetoviciae* TAL-12 support the presence of an expanded light-harvesting repertoire. Canonical regulators of complementary chromatic acclimation, including the RcaE/RcaC two-component system, were not identified in the TAL-12 cyanobacterial MAG. Therefore, although its diversified phycobiliprotein repertoire suggests potential light-harvesting flexibility, the regulatory basis and capacity for chromatic acclimation remain to be experimentally determined. These genomic features are consistent with the environmental context of Talabre Ravine, where cyanobacteria are exposed to strong solar radiation, variable visible light penetration due to sediment and microbial layering, and pronounced diurnal fluctuations in irradiance.

### Comparative phycobiliprotein profiling across Nostocaceae reveals variable accessory pigment modules

To place the phycobiliprotein repertoire of *T. galetoviciae* TAL-12 in a broader evolutionary context, we examined the distribution of phycobiliprotein genes across representative Nostocaceae genomes included in the phylogenomic analysis (Figure 3). This analysis revealed a conserved core set of phycobiliprotein genes across most taxa, including *apcA1*, *apcA3*, *apcB*, *apcB2*, *cpcA*, and *cpcB*. The widespread conservation of these genes indicates that allophycocyanin- and phycocyanin-associated components form a conserved backbone of the phycobilisome system in this group. In contrast, *apcA2* showed a more restricted and lineage-associated distribution. This additional allophycocyanin alpha-subunit gene was present in TAL-12 and in the closely related clade containing the two LEGE 11386 genomes and *Cyanocohniella* sp., while appearing only sporadically in more distant Nostocaceae lineages such as *Trichormus*, *Nostoc*, and *Mojavia* (Figure 3). This pattern suggests that *apcA2* may represent a variable accessory component of the allophycocyanin repertoire, potentially retained or acquired in specific phylogenetic backgrounds.

Genes encoding phycoerythrocyanin subunits (*pecA and pecB*) also displayed a patchy distribution across the tree. These genes were detected in TAL-12, *Cyanocohniella*, one of the LEGE 11386 genomes, and selected representatives of *Nodularia*, *Trichormus*, and *Nostoc*. Notably, the presence of *pecAB* appeared largely mutually exclusive with genes encoding phycoerythrin subunits (*cpeA* and *cpeB*), which were also sporadically distributed among Nostocaceae genomes (Figure 3). This distribution suggests that related Nostocaceae lineages may rely on alternative accessory pigment modules, with some encoding phycoerythrocyanin-associated components and others retaining phycoerythrin-associated modules.

The TAL-12 profile is therefore characterized by the presence of the conserved APC/PC backbone, the lineage-associated *apcA2* gene, and a phycoerythrocyanin module, but by the absence of detectable phycoerythrin biosynthesis genes. Together with its phylogenetic position, genome size, GC content, and ANI values relative to closely related genomes, this pattern supports the view that TAL-12 possesses a distinctive phycobiliprotein repertoire within Nostocaceae. The combination of conserved core components and variable accessory pigment genes highlights diversification of light-harvesting strategies across related cyanobacterial lineages.

### Pigment fractionation supports the genomic absence of phycoerythrin in TAL-12

Comparative genomic analysis showed that the TAL-12 cyanobacterial MAG encodes phycocyanin, allophycocyanin, and phycoerythrocyanin components but lacks detectable *cpeA* and *cpeB* genes encoding canonical phycoerythrin subunits. To evaluate whether a phycoerythrin-like pigment fraction was produced under the culture conditions tested, we performed sequential ammonium sulfate precipitation of phycobiliprotein-containing extracts prepared from cyanobacterial biomass recovered from TAL-12 consortium cultures. The resulting fractions were compared with those obtained from *Nostoc* sp. Caquena CAQ-15, an Andean wetland cyanobacterium from northern Chile previously shown to produce phycoerythrin as its predominant phycobiliprotein and to modulate its pigment composition through complementary chromatic adaptation (Galetovic et al., 2020).

Based on previous laboratory fractionation assays, phycoerythrin-containing pigments are expected to precipitate predominantly in the 0%–35% ammonium sulfate saturation fraction and yield a characteristic pink to purple coloration. This pattern was observed for *Nostoc* sp. CAQ-15 but not for the TAL-12-derived extract (Supplementary Figure S4). No pink or purple phycoerythrin-like fraction was recovered in the 0%–35% saturation range. Instead, the extract produced a blue-pigmented fraction in the 35%–60% saturation range, consistent with phycocyanin enrichment.

Together, the absence of detectable *cpeA* and *cpeB* genes and the absence of a phycoerythrin-like pigment fraction indicate that canonical phycoerythrin production was not detected under the conditions tested. In contrast, recovery of the blue-pigmented fraction supports phycocyanin enrichment. These results are consistent with a phycobiliprotein repertoire comprising phycocyanin-, allophycocyanin-, and phycoerythrocyanin-associated components rather than canonical phycoerythrin.

### Phycocyanin alpha subunit of *Talabrenema galetoviciae* TAL-12 shows sequence features associated with thermostable cyanobacterial phycocyanins

Because pigment fractionation of the TAL-12 consortium-derived extract yielded a blue fraction consistent with phycocyanin enrichment, we next examined the sequence features of the phycocyanin alpha subunit (PCα) from *T. galetoviciae* TAL-12. The PCα amino acid sequence was translated from the Nanopore consensus nucleotide sequence obtained for the targeted PCα amplicon and compared with homologs from thermophilic, thermotolerant, and mesophilic cyanobacteria deposited in GenBank. In the resulting phylogenetic reconstruction, the *T. galetoviciae* TAL-12 PCα sequence grouped within a clade containing several homologs from thermophilic and thermotolerant cyanobacteria, including *Fischerella thermalis*, a filamentous cyanobacterium reported from hot spring environments (Figure 5A). Although some internal nodes showed only moderate bootstrap support, this placement indicates sequence relatedness to PCα homologs from thermally tolerant lineages within the taxonomic sampling included in the analysis. It does not, by itself, establish thermophily or thermal tolerance of *T. galetoviciae* TAL-12.

**FIGURE 5:**
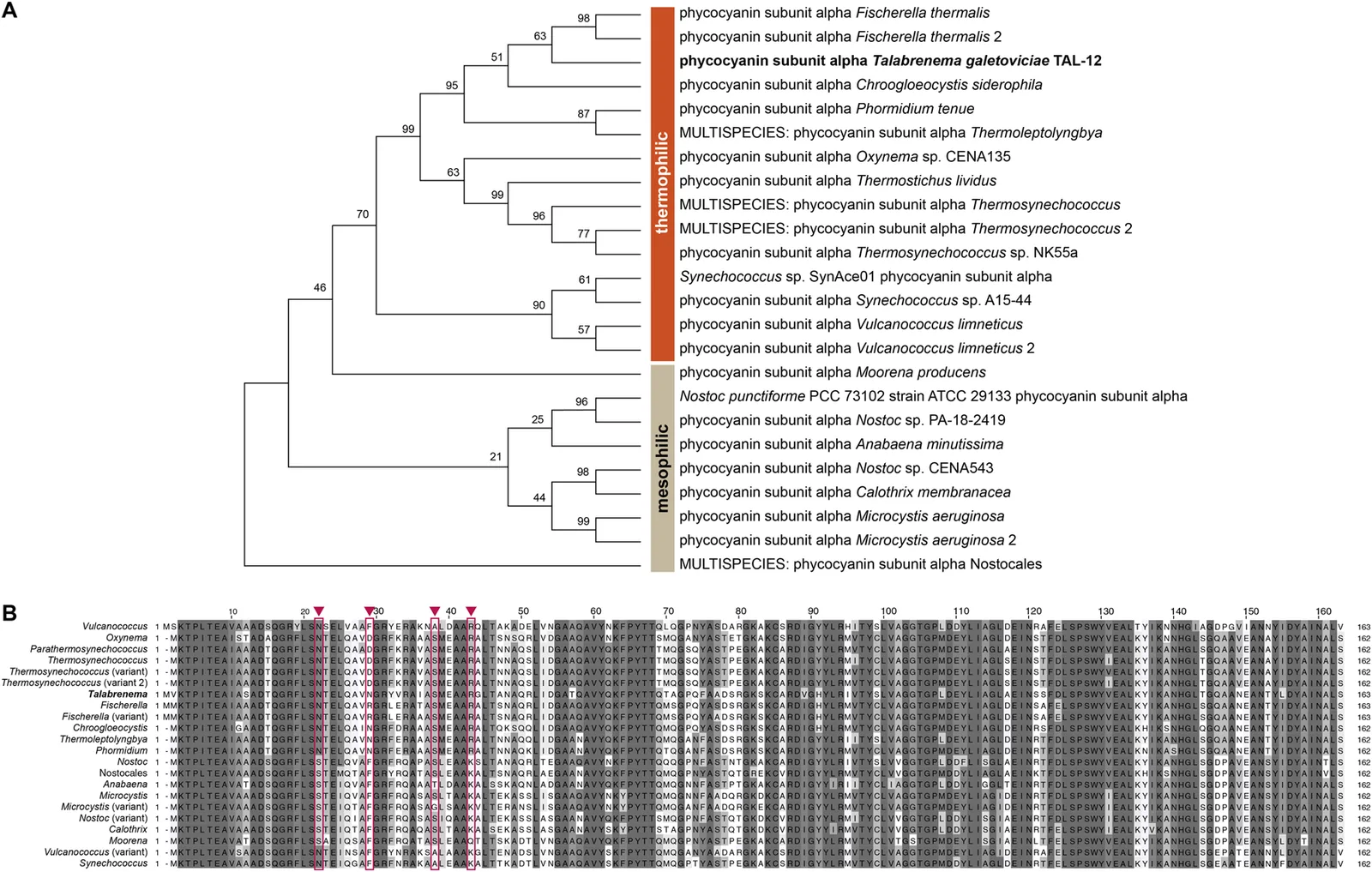
Phylogenetic placement and conserved sequence features of the *Talabrenema galetoviciae* TAL-12 phycocyanin alpha subunit. **(A)** Neighbor-Joining phylogenetic tree based on PCα amino acid sequences from *Talabrenema galetoviciae* TAL-12 and representative thermophilic, thermotolerant, and mesophilic cyanobacteria. The TAL-12 sequence corresponds to the amino acid sequence translated from the Nanopore consensus of the targeted PCα amplicon. The tree was reconstructed in MEGA v11.0.10 using 5,000 bootstrap replicates, and bootstrap support values are shown at the nodes. The TAL-12 PCα sequence grouped within a clade containing several homologs from thermophilic and thermotolerant cyanobacteria, including *Fischerella thermalis*. **(B)** Multiple sequence alignment of the TAL-12 PCα sequence with selected cyanobacterial homologs. Positions corresponding to N21, D28, S37, and R42, previously associated with enhanced phycocyanin thermal stability, are highlighted.

Multiple sequence alignment showed that the *T. galetoviciae* TAL-12 PCα sequence retains several residues previously associated with enhanced thermal stability in cyanobacterial phycocyanins. Residues corresponding to N21, D28, S37, and R42 were conserved or replaced by chemically compatible amino acids in TAL-12 and the thermophilic reference sequences (Figure 5B). These positions have been proposed to contribute to protein stabilization through polar contacts, hydrogen bonding, or electrostatic interactions in phycobiliprotein subunits (Adir et al., 2001; Liang et al., 2018). Their conservation therefore provided a sequence-based rationale for experimentally evaluating the thermal stability of the purified TAL-12 phycocyanin but does not demonstrate their individual contribution to the observed phenotype.

### Phycocyanin purified from TAL-12 consortium cultures shows moderate thermal stability under prolonged heat exposure

Following the sequence-based observations for the *T. galetoviciae* TAL-12 PCα subunit, phycocyanin (PC) was purified from cyanobacterial biomass recovered from TAL-12 consortium cultures and evaluated for stability during prolonged incubation at different temperatures. Purified PC solutions were incubated for up to 72 h at temperatures ranging from 4 °C to 80 °C. Pigment stability was monitored using visible absorption spectra, residual PC concentration, and visual assessment of color retention (Figure 6).

**FIGURE 6:**
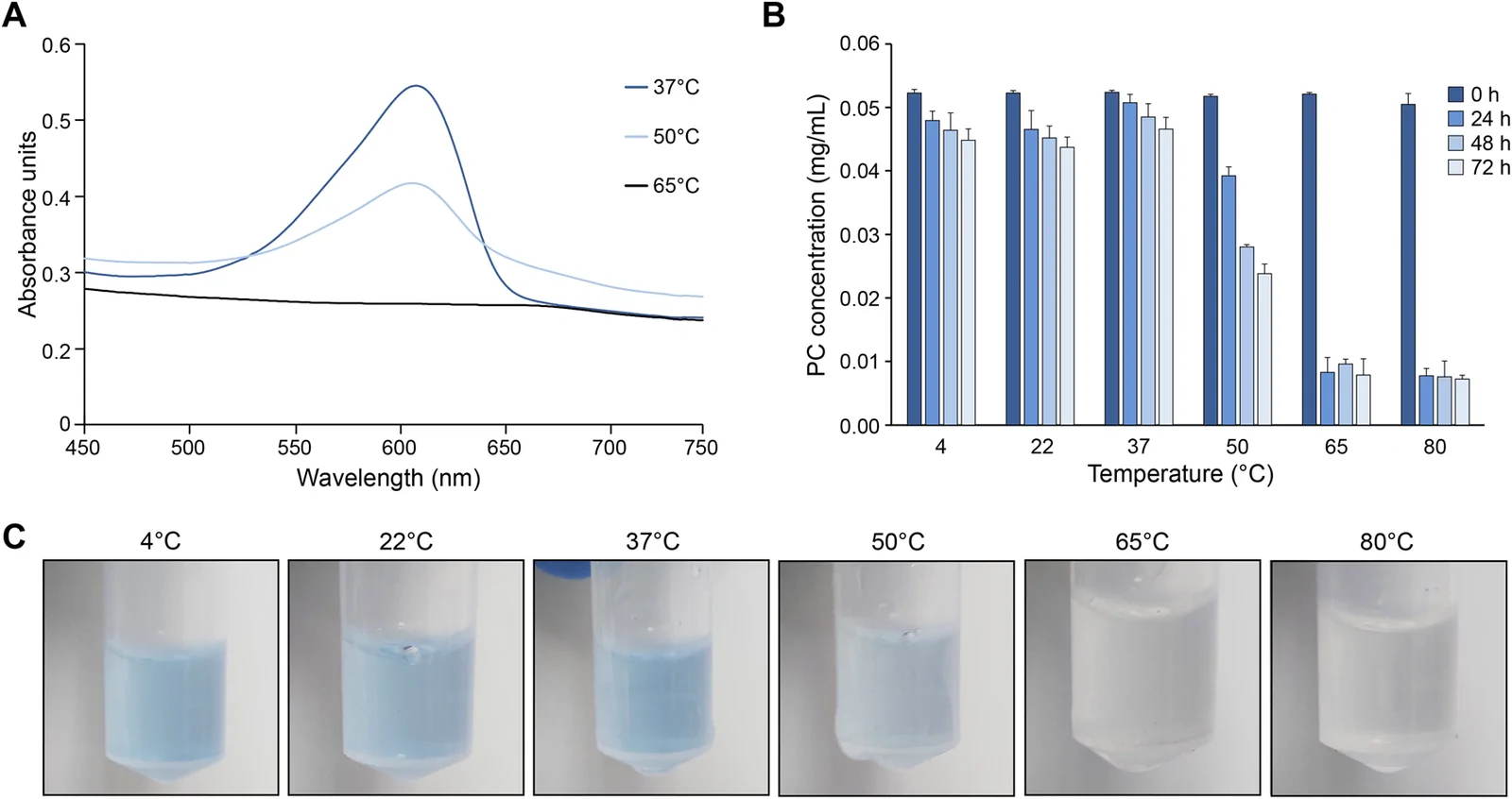
Thermal stability of phycocyanin purified from TAL-12 consortium-derived cyanobacterial biomass. Purified phycocyanin (PC) was incubated at different temperatures for up to 72 h. **(A)** Representative visible absorption spectra showing changes in the characteristic PC absorption profile after incubation at selected temperatures. **(B)** Residual PC concentration after incubation at 4 °C, 22 °C, 37 °C, 50 °C, 65 °C, and 80 °C for 0, 24, 48, and 72 h, calculated from the corresponding absorbance values. Measurements were performed in triplicate. **(C)** Representative appearance of PC solutions after heat exposure, showing progressive loss of blue coloration with increasing temperature. PC showed substantial retention between 4 °C and 37 °C, partial retention at 50 °C, and marked pigment loss at temperatures ≥65 °C.

The purified TAL-12-derived PC retained approximately 84%–92% of its initial concentration after 72 h between 4 °C and 37 °C. At 50 °C, pigment retention decreased progressively: approximately 76% remained after 24 h, whereas approximately half of the initial PC concentration remained after 48–72 h. More pronounced losses were observed at higher temperatures, with only approximately 15% residual PC detected after 48–72 h at 65 °C and 80 °C. Incubation at 65 °C also caused a marked alteration of the characteristic PC absorption profile, consistent with pigment degradation or protein denaturation.

Visual inspection supported the spectrophotometric measurements, showing progressive loss of blue coloration with increasing temperature and incubation time. Together, these results indicate substantial pigment retention during prolonged incubation between 4 °C and 37 °C, partial retention at 50 °C, and marked pigment loss at temperatures ≥65 °C. The purified TAL-12-derived phycocyanin therefore displayed moderate thermal stability under the conditions tested.

### TAL-12 consortium cultures tolerate prolonged exposure to elevated temperature

Because phycocyanin purified from TAL-12 consortium-derived cyanobacterial biomass retained partial stability under moderate heat exposure, we next evaluated the response of TAL-12 consortium cultures to prolonged exposure to elevated temperature. Cultures were incubated at 50 °C for 15 days, whereas control cultures were maintained at 22 °C under standard growth conditions. Chlorophyll concentration was monitored over time as a proxy for cyanobacterial biomass and pigment retention (Supplementary Figure S5).

Exposure to 50 °C caused a progressive decrease in chlorophyll concentration during the first days of incubation, consistent with physiological stress affecting the cyanobacterial component. Nevertheless, chlorophyll remained detectable throughout the assay, and the cultures retained visible pigmentation after prolonged incubation (Supplementary Figure S5). In contrast, control cultures maintained at 22 °C showed a comparatively stable pigment profile under standard growth conditions. Thus, despite the initial decrease in chlorophyll concentration, TAL-12 consortium cultures maintained detectable cyanobacterial pigmentation during sustained exposure to elevated temperature. Together with the moderate thermal stability observed for purified phycocyanin, the maintenance of detectable chlorophyll and visible pigmentation after incubation at 50 °C supports heat-resilience traits at both the pigment level and in the cyanobacterial component of TAL-12 consortium cultures. These features may be relevant to its persistence under the temperature fluctuations characteristic of exposed desert habitats.

### *Talabrenema galetoviciae* TAL-12 encodes a scytonemin biosynthetic gene cluster consistent with UV-photoprotective potential

Given the intense solar and ultraviolet radiation characteristic of the Atacama Desert, we examined whether the TAL-12 cyanobacterial MAG encoded genetic determinants associated with UV photoprotection. Genome analysis identified a putative scytonemin biosynthetic gene cluster located approximately 40 kbp downstream of the *cpc* and *pec* phycobiliprotein regions. The cluster contained homologs of core genes associated with precursor formation, aromatic intermediate condensation, oxidative maturation, and transport-related steps of the scytonemin pathway (Figure 7A).

**FIGURE 7:**
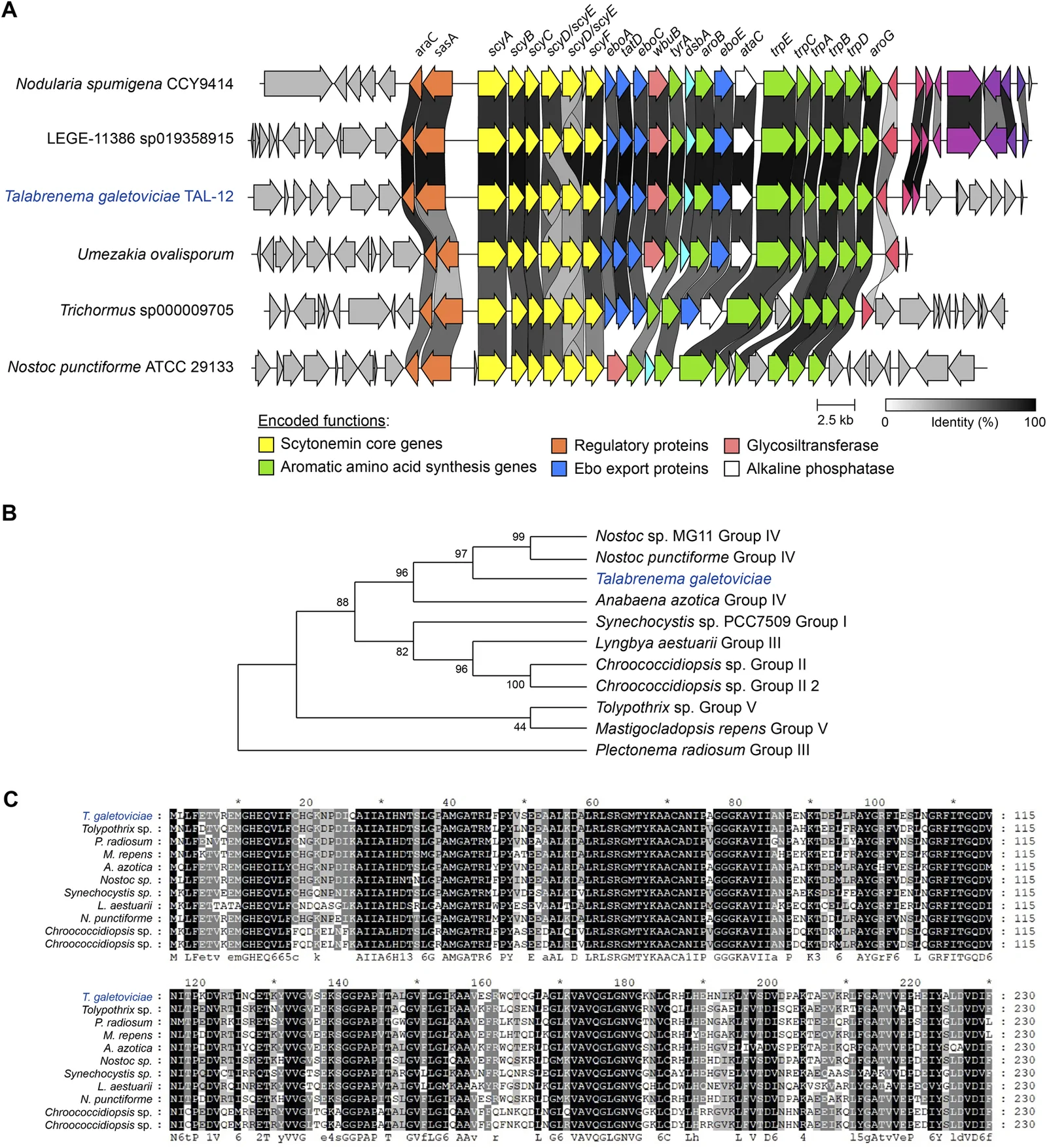
Scytonemin biosynthetic gene cluster organization and ScyB sequence conservation in *Talabrenema galetoviciae* TAL-12 and representative cyanobacteria. **(A)** Comparative genomic organization of the scytonemin biosynthetic region in the TAL-12 cyanobacterial MAG and representative Nostocales genomes, including the canonical scytonemin cluster of *Nostoc punctiforme* ATCC 29133. Genes are shown according to genomic orientation and colored by predicted function, including scytonemin core biosynthesis, aromatic amino acid biosynthesis, regulatory functions, Ebo-associated proteins implicated in precursor export, glycosyltransferase, and alkaline phosphatase. Shading indicates sequence identity between homologous genomic regions. The TAL-12 cyanobacterial MAG retains the principal components of the scytonemin pathway while displaying lineage-specific differences in gene content and organization. **(B)** Neighbor-joining phylogenetic analysis of ScyB amino acid sequences from *Talabrenema galetoviciae* TAL-12 and representative cyanobacteria, reconstructed from the Gblocks-filtered alignment using 5,000 bootstrap replicates. Nodes with bootstrap support ≥70% are indicated by magenta circles, and the scale bar represents 0.1 substitutions per site. The TAL-12 ScyB sequence is highlighted in blue and groups with homologs from filamentous Nostocales-related cyanobacteria. **(C)** Gblocks-filtered multiple sequence alignment of the ScyB sequences included in the phylogenetic analysis, comprising 342 retained amino acid positions and showing extensive sequence conservation across the analyzed homologs.

The *T. galetoviciae* TAL-12 scytonemin gene region retained the principal components of the pathway while displaying differences in gene content and local organization. The region contained genes annotated as components of scytonemin core biosynthesis, aromatic amino acid biosynthesis, and pathway regulation, together with Ebo-associated proteins implicated in precursor export, a glycosyltransferase, and an alkaline phosphatase (Figure 7A). Its organization was broadly similar to scytonemin biosynthetic regions in related Nostocales, including the canonical cluster of *Nostoc punctiforme* ATCC 29133, but showed lineage-specific differences. The specific contribution of the Ebo-associated and accessory proteins in *T. galetoviciae* TAL-12 remains to be experimentally determined.

Phylogenetic analysis placed the *T. galetoviciae* TAL-12 ScyB protein in a supported cluster with the homolog from Nostocales cyanobacterium LEGE 11386, close to ScyB sequences from *Trichormus* sp. and *Nodularia spumigena* (Figure 7B). The Gblocks-filtered alignment retained 342 amino acid positions and showed extensive conservation across TAL-12 ScyB and the analyzed cyanobacterial homologs (Figure 7C), supporting its annotation as a scytonemin biosynthesis protein.

Together, the presence and organization of the scytonemin gene cluster, conservation of core pathway components, and ScyB sequence features support the genomic capacity of *T. galetoviciae* TAL-12 to encode a scytonemin-associated UV-photoprotective pathway. However, scytonemin production, extracellular accumulation, and photoprotective activity were not directly measured.

### No canonical cyanotoxin biosynthetic gene clusters were detected in the TAL-12 cyanobacterial MAG

Because several cyanobacterial lineages, including members of Nostocales, can produce cyanotoxins, we examined the TAL-12 cyanobacterial MAG for biosynthetic determinants associated with microcystin, nodularin, cylindrospermopsin, saxitoxin, and anatoxin-a/homoanatoxin-a. AntiSMASH did not identify biosynthetic gene clusters assigned to any of these cyanotoxin families.

Targeted tBLASTn screening using the panel of 30 reference proteins returned at least one sequence-similarity hit for 23 queries. Alignments were recovered for all six microcystin proteins, all six nodularin proteins, three of the five cylindrospermopsin proteins, two of the six saxitoxin proteins, and six of the seven anatoxin-a/homoanatoxin-a proteins included in the analysis. No hits meeting the search threshold were recovered for CyrA, CyrJ, SxtB, SxtG, SxtI, SxtS, or AnaD. Most of the strong matches involved large non-ribosomal peptide synthetase or polyketide synthase proteins. Moreover, queries from different cyanotoxin pathways frequently aligned to overlapping regions of the same contigs. This pattern was particularly evident among microcystin, nodularin, and cylindrospermopsin proteins and is consistent with similarity to shared catalytic domains rather than the presence of distinct pathway-specific homologs. Isolated matches to shorter or broadly conserved enzymes were likewise not accompanied by the remaining components of their corresponding pathways.

Importantly, none of the five pathways showed a complete and colocalized set of core genes with an organization compatible with a characterized cyanotoxin biosynthetic cluster. Thus, despite the recovery of individual similarity matches, the combined antiSMASH and targeted tBLASTn analyses provided no evidence of canonical biosynthetic gene clusters for microcystin, nodularin, cylindrospermopsin, saxitoxin, or anatoxin-a/homoanatoxin-a in the TAL-12 cyanobacterial MAG.

## Discussion

Microbial persistence in extreme desert environments depends on the adaptive capacity of individual organisms and may also be influenced by interactions within structured communities. The TAL-12 consortium provides a genome-resolved view of an enriched cyanobacteria-associated community recovered from a high-altitude, river-influenced site in the Atacama Desert, where intense solar and ultraviolet radiation, oxidative stress, temperature fluctuations, and metal-rich substrates impose multiple environmental constraints. The TAL-12 cyanobacterial MAG encodes the core machinery for oxygenic photosynthesis, carbon fixation, light harvesting, and pigment biosynthesis, whereas the associated heterotrophic MAGs encode differentiated predicted repertoires related to organic compound turnover, nitrogen and sulfur transformations, iron acquisition, cofactor metabolism, detoxification, and stress responses. Comparable functional partitioning has been proposed for cyanobacterial mats, cyanospheres, and macrocolonies, including the Andean Llayta system, in which a dominant cyanobacterium occurs within a defined microbial neighborhood rather than as an isolated organism (Hube et al., 2009; Paerl and Pinckney, 1996; Stal, 2012; Vilo et al., 2022).

The uneven distribution of predicted metal-, biocide-, oxidative-, and chemical-stress response functions, which were primarily associated with heterotrophic MAGs, is consistent with potential functional complementarity under the multiple environmental pressures characteristic of the Atacama Desert (Azua-Bustos and González-Silva, 2014; Orellana et al., 2018; Schulze-Makuch et al., 2018; Yadav et al., 2022). However, the metagenomic data characterize the final enriched consortium and do not demonstrate that community composition remained unchanged throughout enrichment, that metabolites are exchanged among its members, or that the associated bacteria directly protect the cyanobacterial primary producer. Community-level stress resilience should therefore be considered a hypothesis supported by the observed functional differentiation, rather than a demonstrated emergent property.

Phylogenomic analyses consistently placed *T. galetoviciae* TAL-12 as a distinct lineage within Nostocaceae. Although the taxonomic tools used produced partially conflicting genus-level assignments, its independent phylogenetic position, low ANI values relative to the closest available genomes, and lack of confident assignment to any described genus support its recognition as a novel species and likely a novel genus. Such uncertainty at higher taxonomic ranks is expected for cyanobacterial lineages from underexplored environments, where limited representation of type genomes and incomplete taxon sampling constrain genome-based classification.

The closest phylogenomic relatives of *T. galetoviciae* TAL-12 belong to a poorly characterized radiation of Nostocaceae encompassing aquatic, subaerial, marine-associated, and high-altitude lineages. Its proximity to *Cyanocohniella* sp. LLY, reconstructed from the edible Andean cyanobacterial macrocolony Llayta, is particularly relevant because few cyanobacterial systems from northern Chile have been examined using genome-resolved approaches (Azua-Bustos and González-Silva, 2014; Crits-Christoph et al., 2016; Finstad et al., 2017; Vilo et al., 2022). The proposed name *Talabrenema galetoviciae* reflects the geographic origin of the lineage in Quebrada Talabre and its distinction from currently described Nostocaceae genera. Beyond its taxonomic novelty, the availability of a maintainable consortium dominated by this lineage provides a useful experimental system for investigating the diversification of Nostocaceae in extreme desert-associated habitats.

The phycobiliprotein repertoire of *T. galetoviciae* TAL-12 distinguishes this lineage from several related Nostocaceae. The coexistence of phycocyanin (PC), allophycocyanin (APC), and phycoerythrocyanin (PEC) genes, together with the absence of the canonical phycoerythrin genes *cpeA* and *cpeB*, indicates a phycobilisome system dominated by PC/APC/PEC components. This interpretation is consistent with pigment fractionation, which supported PC enrichment but did not reveal a phycoerythrin-like fraction under the tested culture conditions. The resulting antenna architecture may favor light harvesting across the orange-red spectral range while retaining additional flexibility through PEC and diversified APC components.

Comparative genomic profiling across Nostocaceae revealed a conserved backbone comprising *cpcAB* and core *apc* genes, together with a more variable distribution of accessory modules such as *apcA2*, *pecAB*, and *cpeAB*. The restricted and, in several genomes, apparently mutually exclusive occurrence of *pecAB* and *cpeAB* is consistent with lineage-specific diversification of phycobilisome composition. Because PEC proteins can incorporate phycoviolobilin and phycocyanobilin chromophores and have been associated with CA7-type responses, the presence of *pecAB* may broaden light absorption across the green-orange spectral range and provide an alternative to PE-containing accessory modules. The additional APC paralogs identified in TAL-12 provide a further potential source of functional variation. The dispersed genomic distribution of these paralogs, including the proximity of some copies to mobile-element- or toxin-antitoxin-associated regions, is compatible with a dynamic evolutionary history involving duplication, genome rearrangement, or horizontal acquisition, although these alternatives cannot be distinguished from the available data. Despite their sequence divergence, their predicted structural conservation and retention of key residues argue against their interpretation as degraded remnants. They could instead influence bilin binding, spectral tuning, linker interactions, or phycobilisome assembly, as described for specialized APC variants in other cyanobacteria (Gisriel et al., 2024; Hirose et al., 2019; Kehoe and Gutu, 2006; Mondal et al., 2024; Six et al., 2007; Wang and Chen, 2022). Their specific roles in TAL-12, however, remain to be experimentally established.

The absence of clearly identifiable RcaE/RcaC regulators prevents assignment of *T. galetoviciae* TAL-12 to a canonical chromatic acclimation pathway. Nevertheless, pigment plasticity and light-responsive regulation have been reported in *Nodularia* and other Nostocales even when canonical chromatic acclimation systems are not evident (Voß et al., 2013; Kopf et al., 2015). Accordingly, the PC/APC/PEC repertoire, absence of canonical PE, structurally conserved APC paralogs, and variable distribution of accessory pigment modules support the potential for enhanced chromatic flexibility, but do not demonstrate regulated chromatic acclimation. Transcriptomic and pigment-composition analyses under defined light spectra will be required to determine whether these genomic features produce condition-dependent remodeling of the light-harvesting apparatus of *T. galetoviciae* TAL-12.

The sequence and experimental analyses indicate that TAL-12 phycocyanin possesses moderate, rather than extreme, thermal stability. The PCα subunit contains polar or charged substitutions at positions previously associated with enhanced stabilization of cyanobacterial phycobiliproteins, including changes involving Asn21, Asp28, Ser37, and Arg42. These residues could favor local hydrogen-bonding or electrostatic interactions, providing a plausible molecular contribution to protein stability (Adir et al., 2001; Liang et al., 2018; Pittera et al., 2017; Dagnino-Leone et al., 2022). However, the contribution of individual substitutions remains predictive and will require direct structural or mutational validation.

Consistent with this sequence-based interpretation, purified TAL-12 phycocyanin remained stable during prolonged exposure between 4 °C and 37 °C. At 50 °C, approximately 76% of the initial phycocyanin concentration remained after 24 h, whereas degradation increased substantially during longer exposure and at temperatures of 65 °C or higher. Phycocyanins from established thermophilic cyanobacteria also show high retention at 50 °C–60 °C, although differences in exposure time and experimental conditions prevent direct ranking of their thermal stability relative to TAL-12 (Leu et al., 2013; Liang et al., 2018). Overall, the results support moderate rather than extreme thermal stability. This property may be ecologically relevant in exposed desert habitats subject to temperature fluctuations and supports further investigation of TAL-12 phycocyanin for applications requiring stability under moderate processing or storage conditions.

At the culture level, exposure of TAL-12 consortium cultures to 50 °C for 15 days caused a progressive decrease in chlorophyll relative to cultures maintained at 22 °C, confirming that elevated temperature imposed physiological stress. Nevertheless, chlorophyll remained detectable and cultures retained visible pigmentation throughout the assay, indicating persistence of pigment-containing cyanobacterial biomass during prolonged heat exposure. Under the classical definition of thermophilic cyanobacteria as organisms with optimal growth above 45 °C (Castenholz, 1969), the present results do not demonstrate thermophily because growth optima were not determined. Because chlorophyll retention does not demonstrate active growth or optimal performance at 50 °C, and because the experiment used consortium cultures rather than an axenic *T. galetoviciae* culture, these observations should not be interpreted as evidence that *T. galetoviciae* TAL-12 is thermophilic. Together, the moderate thermal stability of purified phycocyanin and the maintenance of chlorophyll and visible pigmentation in consortium cultures support heat-resilience traits at both the protein and culture levels.

Scytonemin provides a complementary form of pigment-based protection to the intracellular phycobiliprotein light-harvesting system. This extracellular or sheath-associated pigment predominantly attenuates incident UV radiation before it reaches sensitive cellular targets, providing passive and persistent photoprotection once deposited in peripheral cell structures (Garcia-Pichel and Castenholz, 1991; Gao and Garcia-Pichel, 2011; Gao et al., 2021). The presence of a Nostocales-like scytonemin biosynthetic gene cluster in the TAL-12 cyanobacterial MAG is therefore consistent with the environmental conditions of Quebrada Talabre, where high altitude, intense irradiance, reflective substrates, desiccation, and oxidative stress impose strong photoprotective demands. Conservation of the principal biosynthetic components supports the predicted capacity for scytonemin biosynthesis, although pigment production under the tested culture conditions was not experimentally demonstrated.

The *T. galetoviciae* TAL-12 scytonemin genomic region also contains Ebo-associated genes. In many cyanobacteria, these genes occur within the scytonemin operon, whereas in *Nostoc punctiforme* they are located at a separate genomic locus. Mutational studies in *N. punctiforme* indicate that the Ebo proteins are required for translocation of the scytonemin monomer from the cytoplasm to the periplasm, where subsequent oxidative maturation occurs (Klicki et al., 2018). Their conservation therefore supports a potential role in precursor export and subsequent extracellular pigment localization, although this function remains to be experimentally confirmed in *T. galetoviciae* TAL-12. The placement of TAL-12 ScyB among homologs from filamentous Nostocales-related lineages further indicates that this pathway reflects a Nostocales-like photoprotective ancestry rather than the thermophile-associated pattern observed for PCα. Thus, *T. galetoviciae* TAL-12 combines two evolutionarily distinct pigment-related systems: a diversified phycobiliprotein repertoire potentially contributing to light-harvesting flexibility and a scytonemin pathway consistent with passive UV protection in a high-radiation desert environment.

The pigment repertoire of *T. galetoviciae* TAL-12 supports its potential relevance for extremophile-based biotechnology. Phycobiliproteins are widely investigated as natural colorants, fluorescent probes, and bioactive compounds, whereas scytonemin has attracted interest as a chemically stable natural UV-screening molecule for photoprotective materials, cosmetic formulations, and biomaterials (Spolaore et al., 2006; Sonani, 2016; Dumay and Morançais, 2016; Gao et al., 2021; Liu et al., 2022; Fernández-Rojas et al., 2026). The recovery of phycocyanin-rich fractions with moderate thermal stability, together with the genome-encoded potential for scytonemin biosynthesis, identifies *T. galetoviciae* TAL-12 as a candidate for further pigment-oriented investigation. However, its applied value will depend on experimental determination of pigment yield, production scalability, fluorescence, pH and photochemical stability, bioactivity, formulation performance, and process compatibility. Scytonemin production and localization must also be confirmed before its biotechnological potential can be evaluated.

Safety assessment is essential before developing cyanobacterial biomass or extracts for applied use, particularly among Nostocales, which include cyanotoxin-producing lineages. The absence of canonical biosynthetic gene clusters for microcystin, nodularin, cylindrospermopsin, saxitoxin, and anatoxin-a/homoanatoxin-a in the TAL-12 cyanobacterial MAG provides an initial genomic indication of reduced risk for these major cyanotoxin families. This result is consistent with previous safety-oriented studies of northern Chilean cyanobacteria and Llayta-associated *Cyanocohniella*, which illustrate the importance of combining genomic and chemical approaches when evaluating cyanobacterial biomass (Galetovic et al., 2020; Vilo et al., 2022). Nevertheless, the absence of recognized cyanotoxin clusters does not establish complete innocuity, because uncharacterized secondary metabolites or culture-dependent bioactivities may remain undetected. Biochemical toxin screening, metabolomic profiling, and cytotoxicity assays will therefore be required before considering TAL-12 consortium biomass or derived pigments and extracts for food, cosmetic, nutraceutical, or pharmaceutical applications.

Overall, this study resolves two connected levels of cyanobacterial adaptation in an extreme desert-associated system. At the community level, the TAL-12 consortium comprises a dominant cyanobacterial primary producer and phylogenetically diverse heterotrophic members with differentiated predicted metabolic and stress-response repertoires. At the organism level, *T. galetoviciae* TAL-12 represents a distinct Nostocaceae lineage combining a diversified phycobiliprotein system, moderately thermostable phycocyanin, and a Nostocales-like scytonemin biosynthetic pathway. Complementary culture-level assays showed that the cyanobacterial component retained detectable chlorophyll and visible pigmentation during prolonged heat exposure. These findings establish a genomic and experimental framework for investigating how photobiological specialization, heat resilience, UV photoprotection, and potential community-level functional complementarity contribute to persistence in desert-associated habitats. They also identify *T. galetoviciae* TAL-12 as a promising, but still incompletely characterized, source of pigment-related traits for extremophile-based biotechnology.

Several limitations define the scope of these conclusions. Metagenomic sequencing characterized the final enriched TAL-12 consortium at a single stage and did not determine whether community composition remained unchanged during the successive enrichment treatments or subsequent long-term cultivation. Genome annotation identifies predicted functional potential but does not demonstrate metabolic activity, metabolite exchange, or direct protection of the cyanobacterial member by associated bacteria. Likewise, the present data do not establish regulated chromatic acclimation, the functions of individual phycobiliprotein paralogs, scytonemin production, active cyanobacterial growth at 50 °C, or the complete absence of toxic bioactivity. Longitudinal community profiling, reduced-consortium and co-culture experiments, transcriptomic and pigment analyses under defined light and temperature conditions, direct scytonemin detection, growth and viability measurements, metabolomics, and biochemical safety assays will be required to test these predictions and determine which traits are constitutive or condition-dependent.

## Data Availability

The metagenome-assembled genomes generated in this study were deposited in the NCBI database under the BioProject accession number PRJNA1529252. Additional data supporting the findings of this study are available from the corresponding authors upon reasonable request.
