# Supplementary material for: A genome-resolved cyanobacterial consortium from the Atacama Desert reveals novel bacterial diversity, stress-resilience potential, chromatic flexibility, and photoprotection
Source: Front Bioeng Biotechnol. 2026 Sep 25;14:1939861. doi: 10.3389/fbioe.2026.1939861 (PMC13649963; doi:10.3389/fbioe.2026.1939861)
Supplement: Supplementary file 1 [file DataSheet1.pdf]

A genome-resolved cyanobacterial consortium from the Atacama Desert reveals novel bacterial diversity, stress-resilience potential, chromatic flexibility, and photoprotection

Alexandra Galetović[1,3], Jan Ortiz[1,3], Gabriel Peña[1,3], Patricio Arros[2], Nicolás Guerra-Leal[2], Camilo Berríos-Pastén[2], Manuel Silva[2], Joaquín Acosta[2], Macarena A. Varas[4], Miguel L. Allende[3,*], Andrés E. Marcoleta[2,*].

[1]Laboratorio de Genómica Microbiana, Unidad Bioquímica, Departamento Biomédico, Facultad de Ciencias de la Salud, Universidad de Antofagasta. Antofagasta 1270300, Chile.

[2]Grupo de Microbiología Integrativa, Facultad de Ciencias, Universidad de Chile. Santiago 7800003, Chile.

[3]Millennium Institute Center for Genome Regulation, Facultad de Ciencias, Universidad de Chile. Santiago 7800003, Chile.

[4]Vicerrectoría de Investigación y Postgrado, Universidad de La Serena. La Serena 1700000, Chile

*Corresponding authors: amarcoleta@uchile.cl, mallende@uchile.cl

SUPPLEMENTARY MATERIAL

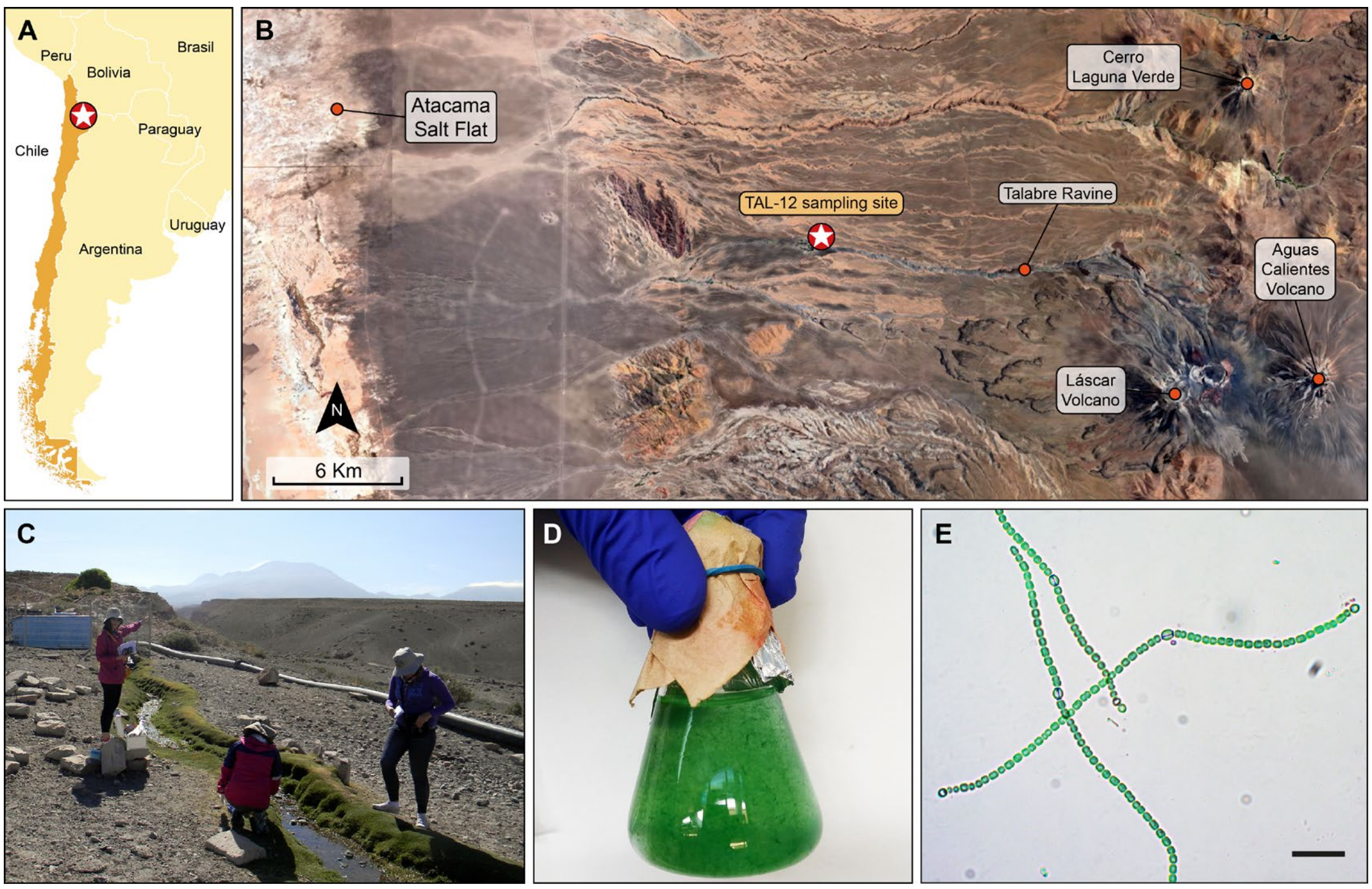

Figure S1. Geographic location, sampling habitat, and representative culture of the enriched TAL-12 consortium. (A) Location of the TAL-12 sampling region in northern Chile. (B) Satellite view showing the TAL-12 sampling site in the Talabre Ravine (23°18′56.1″S, 67°53′10.7″W), within the Atacama Salt Flat basin and near the Láscar and Aguas Calientes volcanoes. The sampling site is located at approximately 3,300 m above sea level. (C) Field view of the river-influenced habitat during sample collection. (D) Representative culture of the enriched TAL-12 consortium. (E) Bright-field micrograph showing the dominant filamentous cyanobacterial morphotype in the enriched consortium. Scale bar, 20 µm. Satellite imagery in panel B was obtained from Google Earth.

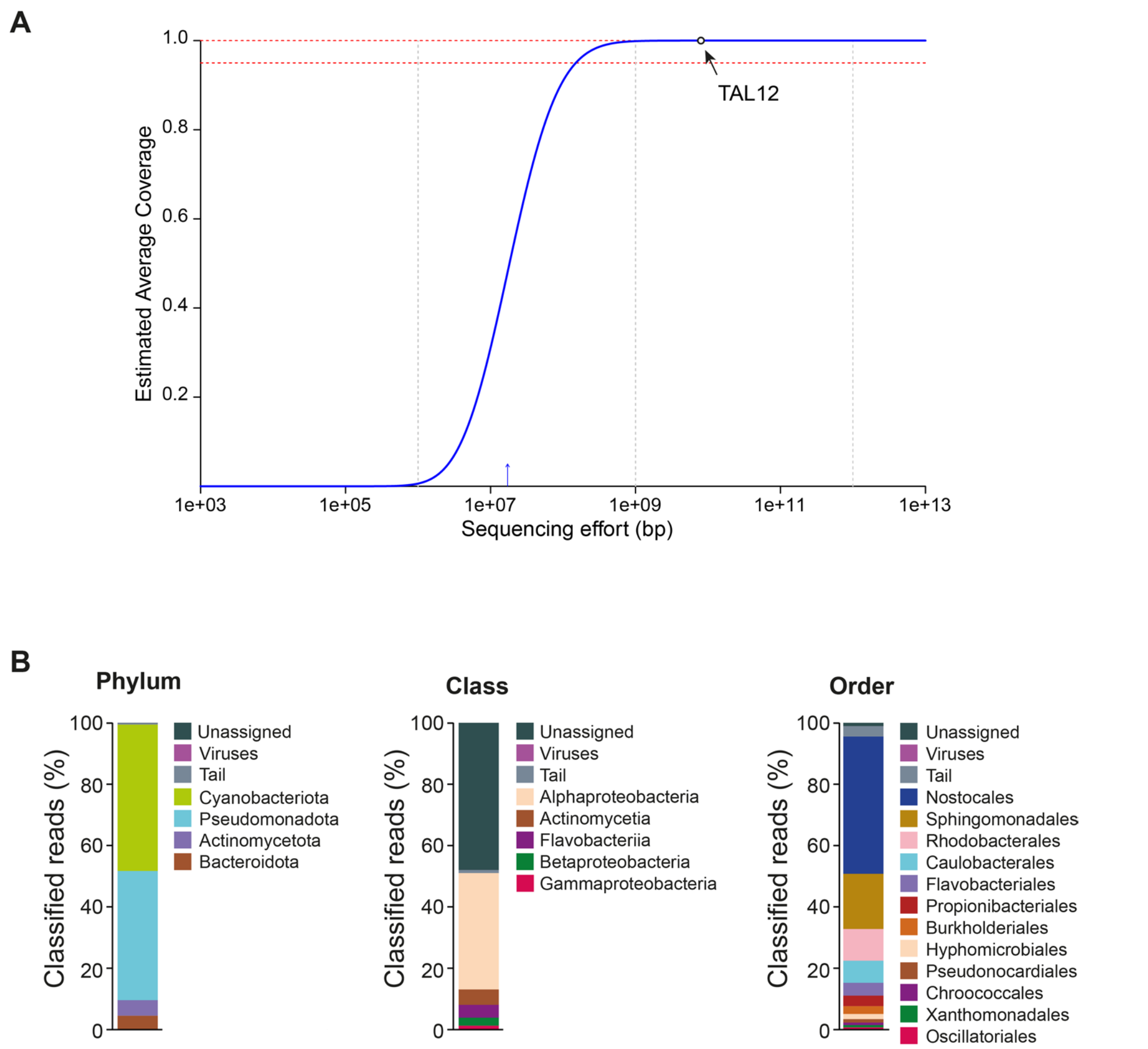

Figure S2. Sequencing effort and taxonomic overview of the TAL-12 metagenome. (A) Nonpareil analysis of the TAL-12 metagenomic dataset, showing the fitted redundancy curve and the estimated sequencing coverage based on read redundancy. (B) Taxonomic classification of metagenomic reads using Kaiju, displayed at the phylum, class, and order levels.

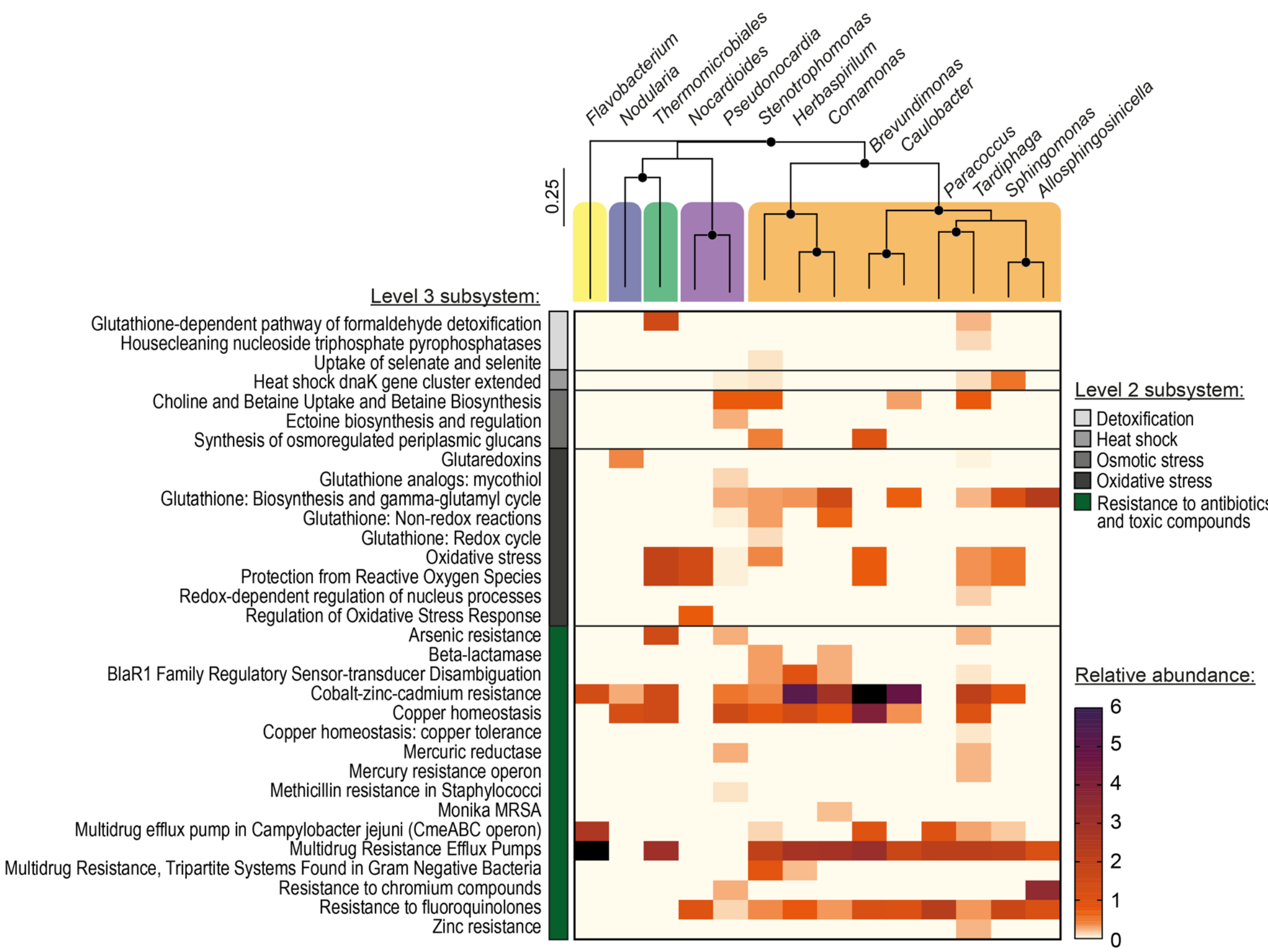

Figure S3. Predicted stress-response functions in TAL-12 consortium genomes based on SUPERFOCUS annotation. Heatmap showing the distribution of SEED subsystem level-3 functional categories across the reconstructed metagenome-assembled genomes (MAGs) from the TAL-12 consortium. Colors represent the relative abundance of genes assigned to each functional category within each MAG. Functional annotations were generated using SUPERFOCUS, and MAGs are ordered according to their taxonomic classification.

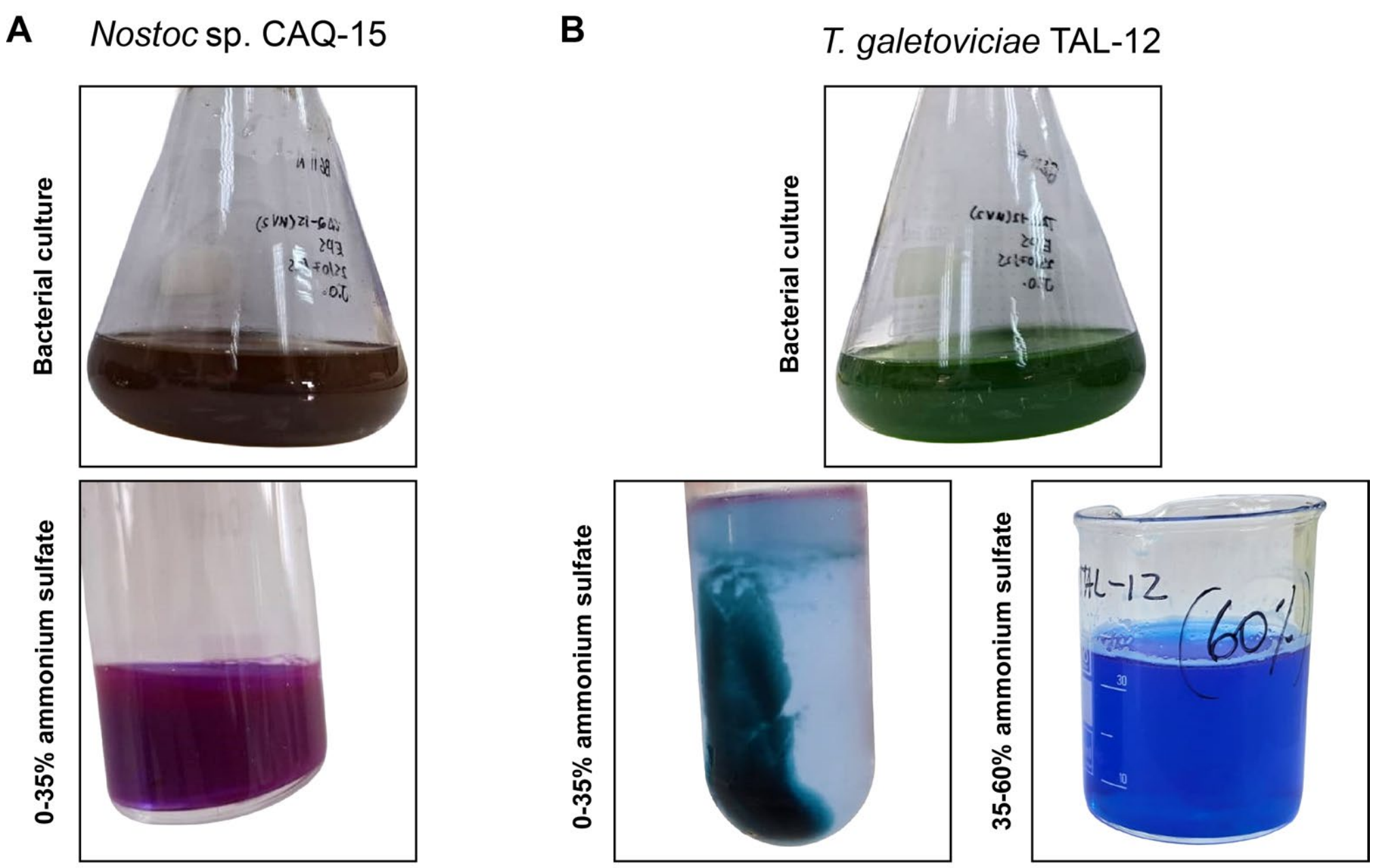

Figure S4. Ammonium sulfate fractionation of phycobiliprotein-enriched extracts from the TAL-12 consortium. Pigment-containing fractions obtained after sequential ammonium sulfate precipitation were used to compare phycoerythrin- and phycocyanin-associated pigment enrichment in TAL-12 and the phycoerythrin-producing control strain *Nostoc* sp. CAQ-15. (A) *Nostoc* sp. CAQ-15 culture and 0–35% ammonium sulfate fraction, showing the characteristic pink/purple coloration expected for phycoerythrin-enriched pigments. (B) Enriched TAL-12 consortium culture and ammonium sulfate fractions. TAL-12 did not yield a pink/purple PE-like fraction in the 0–35% saturation range, but produced a blue-pigmented fraction in the 35–60% saturation range, consistent with phycocyanin enrichment. These results support the genomic prediction that TAL-12 lacks canonical phycoerythrin biosynthesis genes while producing phycocyanin-associated pigments.

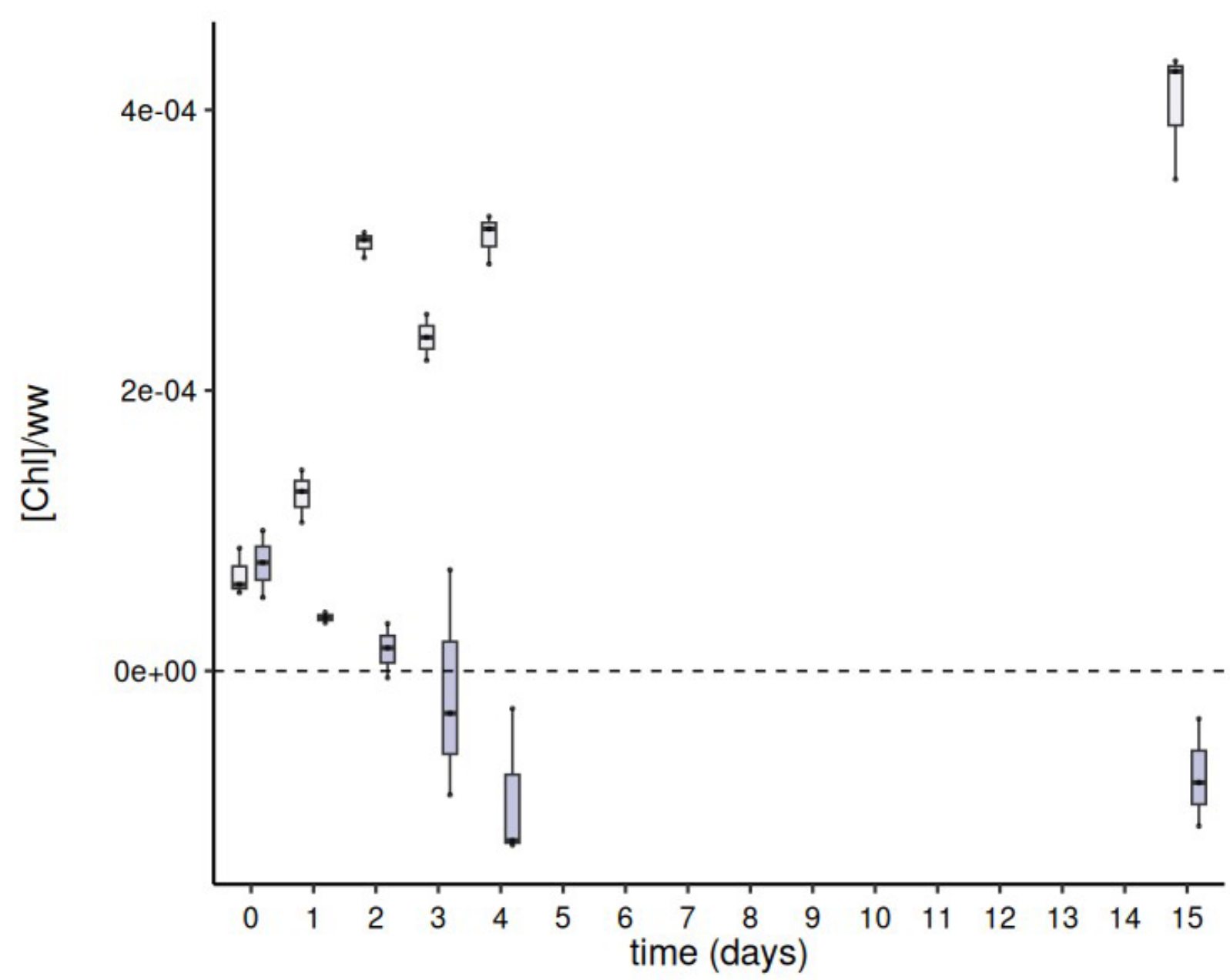

Figure S5. Chlorophyll retention in TAL-12 consortium cultures during prolonged thermal exposure. TAL-12 consortium cultures were incubated at 50°C for 15 days (blue bars), while control cultures were maintained at 22°C (light grey bars). Chlorophyll content was measured at selected time points as a proxy for cyanobacterial biomass and pigment retention. Ww: wet weight. Cultures exposed to 50°C showed a reduction in chlorophyll content during the first days of incubation but retained detectable chlorophyll after prolonged exposure, indicating persistence of pigment-containing cyanobacterial biomass under elevated temperature. These measurements do not demonstrate viability or active growth at 50°C.

Table S1. Reference protein sequences used for phylogenetic analyses of TAL-12 PCα and ScyB. List of reference amino acid sequences used for phylogenetic reconstruction of the phycocyanin alpha subunit (PCα) and the scytonemin biosynthesis protein ScyB from *Talabrenema galetoviciae* TAL-12. The table includes the protein designation, accession number, source taxon, reference category, and relevant notes for each sequence.

Protein	Accession	Taxonomic name	Reference category	Notes
PCα	WP_099799462.1	*Thermostichus lividus*	Thermophilic/reference cyanobacterium	
PCα	WP_180962409.1	*Fischerella thermalis*	Thermophilic/reference cyanobacterium	Paralog/variant 1
PCα	WP_180974923.1	*Fischerella thermalis*	Thermophilic/reference cyanobacterium	Paralog/variant 2
PCα	WP_149818203.1	*Thermosynechococcus sp.*	Thermophilic/reference cyanobacterium	Paralog/variant 1
PCα	WP_011057793.1	*Thermosynechococcus sp.*	Thermophilic/reference cyanobacterium	Paralog/variant 2
PCα	WP_024124992.1	*Thermosynechococcus sp.*	Thermophilic/reference cyanobacterium	
PCα	WP_094591910.1	*Vulcanococcus limneticus*	Thermophilic/reference cyanobacterium	Paralog/variant 1
PCα	WP_094590141.1	*Vulcanococcus limneticus*	Thermophilic/reference cyanobacterium	Paralog/variant 2
PCα	WP_223976655.1	Nostocales cyanobacterium	Mesophilic/reference cyanobacterium	
PCα	WP_103138956.1	*Nostoc sp.*	Mesophilic/reference cyanobacterium	
PCα	WP_138504629.1	*Nostoc sp.*	Mesophilic/reference cyanobacterium	
PCα	WP_190620736.1	*Calothrix membranacea*	Mesophilic/reference cyanobacterium	
PCα	WP_002780314.1	*Microcystis aeruginosa*	Mesophilic/reference cyanobacterium	Paralog/variant 1
PCα	WP_186514662.1	*Synechococcus sp.*	Mesophilic/reference cyanobacterium	
PCα	WP_191129665.1	*Phormidium tenue*	Mesophilic/reference cyanobacterium	
PCα	WP_071103963.1	*Moorena producens*	Mesophilic/reference cyanobacterium	
PCα	WP_200665132.1	*Oxynema sp.*	Mesophilic/reference cyanobacterium	
PCα	WP_190698557.1	*Anabaena minutissima*	Mesophilic/reference cyanobacterium	
PCα	WP_073549092.1	*Chroogloeocystis siderophila*	Mesophilic/reference cyanobacterium	
PCα	WP_103113198.1	*Microcystis aeruginosa*	Mesophilic/reference cyanobacterium	Paralog/variant 2
ScyB	MBE9051210.1	*Nostocales cyanobacterium LEGE 11386*	Reference cyanobacterium	
ScyB	MBW4557638.1	*Trichormus sp. ATA114KO1*	Reference cyanobacterium	
ScyB	WP_272055885.1	*Nodularia spumigena*	Reference cyanobacterium	
ScyB	WP_271919687.1	*Cyanophyceae (multispecies)*	Reference cyanobacterium	
ScyB	MCG6133457.1	*Nostoc sp. LLA-1*	Reference cyanobacterium	
ScyB	WP_273763185.1	*Nostoc sp. UHCC-0926*	Reference cyanobacterium	
ScyB	WP_193917172.1	*Desmonostoc muscorum*	Reference cyanobacterium	
ScyB	WP_194004329.1	*aff. Roholtiella sp. LEGE 12411*	Reference cyanobacterium	
ScyB	MBW4689490.1	*Komarekiella atlantica HA4396MV6*	Reference cyanobacterium	
ScyB	BAZ31295.1	*Cylindrospermum sp. NIES4074*	Reference cyanobacterium	
ScyB	WP_015155131.1	*Chroococcidiopsis (multispecies)*	Reference cyanobacterium	
ScyB	PMB23077.1	*Fischerella thermalis CCMEE-5319*	Thermophilic/reference cyanobacterium	
